# Identifying Beneficial and Adverse Co^4+^ Species in Cobalt‐Based Oxygen Evolution Catalysts via Precursor Polymorphism Engineering

**DOI:** 10.1002/advs.202509145

**Published:** 2025-09-29

**Authors:** Zhongheng Li, Zheng Shu, Lun Li, Wendi Zhang, Jiaqian Kang, Chengcheng Zhong, Ziwen Feng, Jinxian Feng, Hou Ian, Hui Pan

**Affiliations:** ^1^ Institute of Applied Physics and Materials Engineering University of Macau Taipa Macau SAR 999078 China

**Keywords:** Co⁴⁺ species, precursor engineering, oxygen evolution reaction, reaction mechanisms, surface reconstruction

## Abstract

The oxygen evolution reaction (OER) is a critical bottleneck in water electrolysis for hydrogen production, necessitating catalysts that optimize both efficiency and cost. Cobalt‐based materials offer a viable alternative to noble metals, but their development is complicated by uncertainties regarding the role of Co⁴⁺ species formed during operation. Conflicting studies debate whether CoO_2_ acts as the active phase or if Co⁴⁺ species may suppress reactivity. To address this, the Co⁴⁺ impact on OER activity is systematically investigated by tailoring the reconstruction of polymorphic cobalt oxysulfide. Through thermal annealing, crystallinity and local coordination are controlled to selectively stabilize γ‐CoO_2_ and β‐CoO_2_ phases under OER conditions, respectively. Structural analysis reveals that H_2_O/OH^−^ intercalation drives lattice expansion, favoring γ‐CoO_2_ formation, while rigid Co─O bonds limit flexibility, yielding β‐CoO_2_. Mechanistic studies show γ‐CoO_2_ promotes superoxide (Co─O─O─Co) intermediates via the oxygen pathway mechanism (OPM), whereas β‐CoO_2_ follows the conventional adsorbate evolution mechanism (AEM). As a result, γ‐CoO_2_ exhibits superior catalytic performance, with lower overpotentials and enhanced long‐term stability. These insights highlight the pivotal role of Co⁴⁺ micro‐environments in OER performance, offering a rational framework for optimizing transition‐metal catalysts.

## Graphical abstract

This study resolves conflicting roles of Co⁴⁺ species in cobalt‐based oxygen evolution reaction (OER) catalysts by engineering precursor polymorphism. Through controlled annealing of cobalt oxysulfide, researchers selectively stabilize γ‐CoO_2_ (enabling oxygen pathway mechanism) and β‐CoO_2_ (following conventional mechanism). γ‐CoO_2_ demonstrates superior performance, highlighting micro‐environment design for efficient catalysis.

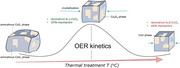

## 1 Introduction

Efficient water electrolysis for hydrogen production is limited by the sluggish kinetics of the oxygen evolution reaction (OER).^[^ ^1^ ^]^ Although noble metal‐based catalysts like iridium and ruthenium oxides demonstrate superior OER performance, their prohibitive cost and scarcity limit industrial scalability.^[^ ^2^, ^3^, ^4^, ^5^ ^]^ This has driven significant research efforts toward earth‐abundant alternatives, with cobalt‐based catalysts emerging as particularly promising due to their favorable combination of natural abundance, cost‐effectiveness, and comparable catalytic performance to noble metals.^[^ ^6^, ^7^, ^8^, ^9^, ^10^, ^11^, ^12^, ^13^ ^]^

Optimizing cobalt‐based catalysts for OER hinges on understanding their structural evolution during operation, particularly the role of metastable Co⁴⁺ species.^[^ ^9^, ^14^, ^15^ ^]^ Despite their presumed importance, the catalytic impact of these species remains contentious. In situ Raman spectroscopy studies indicate CoO_2_ formation under OER conditions, suggesting it may be the active phase.^[^ ^9^, ^10^, ^14^, ^15^, ^16^ ^]^ Conversely, Frei et al. found that Co⁴⁺═O species could hinder reaction kinetics, challenging the notion that higher oxidation states universally improve activity.^[^ ^17^ ^]^ Additionally, Sun et al. highlighted that Co⁴⁺ can manifest in diverse forms, each arising from distinct reconstruction pathways.^[^ ^14^ ^]^ These conflicting findings underscore the need to clarify which Co⁴⁺ species enhance or impair OER performance and to elucidate the mechanisms driving these differences. A systematic comparison of OER activity across well‐defined Co⁴⁺ species is thus essential to resolve these discrepancies and guide catalyst design.

While contemporary strategies focus on the direct manipulation of a catalyst's atomic and electronic structure through chemical, defect,^[^ ^22^, ^23^ ^]^ or strain engineering,^[^ ^24^, ^25^ ^]^ the inherent instability of Co⁴⁺ under ambient conditions necessitates its electrochemical formation during OER, where Co^3^⁺ is oxidized to Co⁴⁺.^[^ ^26^, ^27^ ^]^ Tailoring the local coordination and electronic structure of Co^3^⁺‐rich pre‐catalyst enables the generation of diverse Co⁴⁺ chemical environments. Cobalt oxysulfide is an ideal system for such studies due to its structural polymorphism, allowing varied crystalline forms with identical compositions but distinct atomic arrangements,^[^ ^7^, ^8^, ^22^, ^28^, ^29^, ^30^ ^]^ and its ability to undergo potential‐driven in situ reconstruction into Co⁴⁺ species.^[^ ^10^, ^31^, ^32^ ^]^ Selective polymorph synthesis can be achieved by adjusting parameters such as temperature,^[^ ^33^, ^34^ ^]^ pressure,^[^ ^35^ ^]^ reaction duration,^[^ ^36^ ^]^ and precursor chemistry.^[^ ^37^ ^]^ Among these, thermal treatment is a versatile and precise method, leveraging controlled heating to induce phase transitions and stabilize metastable structural variants, thereby creating unique microchemical environments for Co⁴⁺.^[^ ^24^ ^]^ This approach establishes a foundation for systematically correlating Co⁴⁺ local coordination with OER performance metrics.

**Figure 1:**
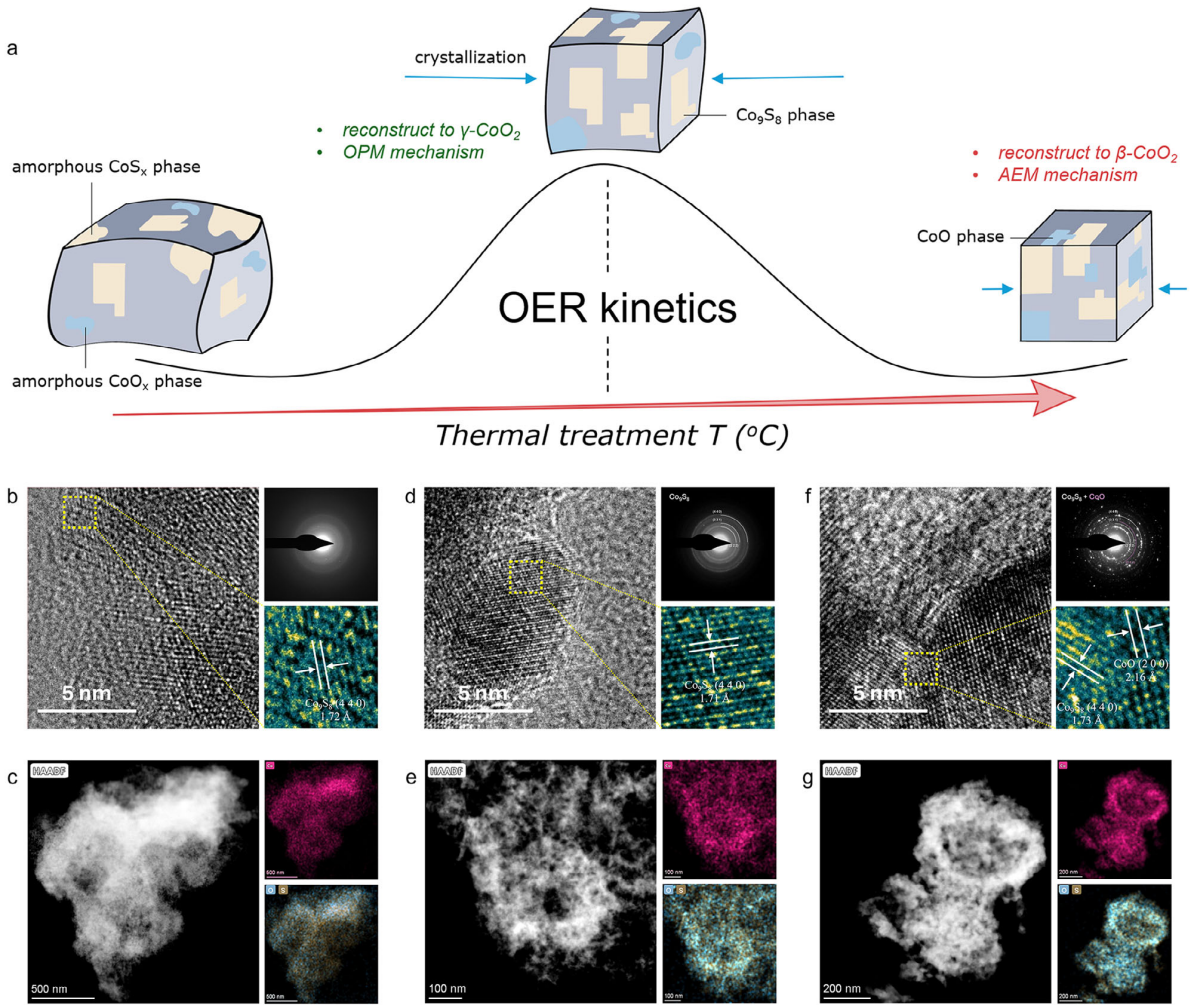
Design philosophy for pre‐catalyst. a) Schematic illustration of the annealing effect on OER. HRTEM images and corresponding SAED pattern of b) PM‐250, d) PM‐350, and f) PM‐450. HAADF‐STEM image and corresponding EDS elemental mappings of c) PM‐250, e) PM‐350, and g) PM‐450.

In this study, we fine‐tuned the annealing conditions of cobalt oxysulfide to selectively form γ‐CoO_2_ and β‐CoO_2_ phases during OER. By precisely controlling temperature, we modulated the crystallinity and local Co^3^⁺ coordination in the pre‐catalyst, guiding its reconstruction into distinct Co⁴⁺ active species (**Figure** **1**a). In situ Raman spectroscopy revealed that flexible Co─O bonds in Co^3^⁺ enable H_2_O/OH^−^ intercalation, expanding interlayer spacing and favoring γ‐CoO_2_ formation. Conversely, rigid Co─O bonds restrict lattice expansion, leading to β‐CoO_2_. In situ attenuated total reflectance infrared (ATR‐IR) spectroscopy further showed that γ‐CoO_2_ promotes superoxide (Co─O─O─Co) intermediates, driving OER via oxygen pathway mechanism (OPM), while β‐CoO_2_ stabilized by Co⁴⁺═O species follows the conventional adsorbate evolution mechanism (AEM). Consequently, the γ‐CoO_2_‐dominated sample exhibited superior OER performance, with low overpotentials (354 mV at 100 mA cm^−^^2^ and 391 mV at 200 mA cm^−^^2^) and robust stability (over 200 h at 100 and 500 mA cm^−^^2^).

## 2 Results and Discussion

### 2.1 Design Principle for Pre‐Catalysts

The pre‐catalyst was prepared through a one‐step solid‐phase sulfur‐exchange reaction by mixing ZIF‐67 metal–organic framework with thioacetamide solution at 120 °C for 4 h (Figures S1 and S2, Supporting Information).^[^ ^29^ ^]^ Then the structural properties of the as‐synthesized polymorphous oxysulfide pre‐catalyst (PM‐P) can be manipulated through thermal treatment in an inert atmosphere (Figure 1a), which facilitates the distinct and gradual crystallization of both the sulfide and oxide phases. The annealing temperature was set at 150, 25, 35, 45, 55, and 700 °C, respectively. Sample annealed at a certain T is abbreviated as PM‐T.

The PM‐P shows a well‐defined hollow rhombic dodecahedral morphology, as indicated by inner cavities in some cracked particles (Figure S2, Supporting Information). X‐ray diffraction (XRD) was performed to examine the temperature effect on the crystallization (Figure S4, Supporting Information). As annealing temperature rises, the characteristic XRD peaks of Co_9_S_8_ intensify, with the crystalline Co_9_S_8_ phase predominating at 350 °C. PM‐350 exhibits broad, weak XRD peaks, suggesting a long‐range disordered structure. Above 450 °C, the crystalline CoO phase emerges, with sharper peaks indicating improved long‐range order.

Transmission electron microscopy (TEM) revealed that the pre‐catalysts consist of spherical nanoparticles, with grain size increasing with annealing temperature (Figure S5, Supporting Information). High‐resolution TEM (HRTEM) and selected area electron diffraction (SAED) highlighted the structural difference across samples. PM‐250 is composed of ordered and disordered lattice fringes, reflecting low crystallinity. A 1.72 Å interplanar spacing corresponds to the Co_9_S_8_ (440) plane, and diffused SAED rings indicate short‐range ordering. PM‐350 displays a similar Co_9_S_8_ (440) plane spacing of 1.71 Å, but shows enhanced crystallinity as evidenced by clear SAED spots, which are attributed to the (400), (331), and (222) planes. In contrast, PM‐450 reveals coexisting Co_9_S_8_ and CoO phases as the lattice fringes show the spacings of 1.73 and 2.16 Å for Co_9_S_8_ (440) and CoO (200), respectively, and a new SAED pattern corresponding to the CoO (200) plane appears. The increased brightness of SAED rings across all samples confirms improved crystallinity at higher temperatures. Additionally, high‐angle annular dark‐field scanning TEM (HAADF‐STEM) images and elemental mappings (Figure1c,e,g) confirm the homogeneous distribution of Co, S, and O in all samples.

### 2.2 Chemical Structure for Pre‐Catalysts

**Figure 2:**
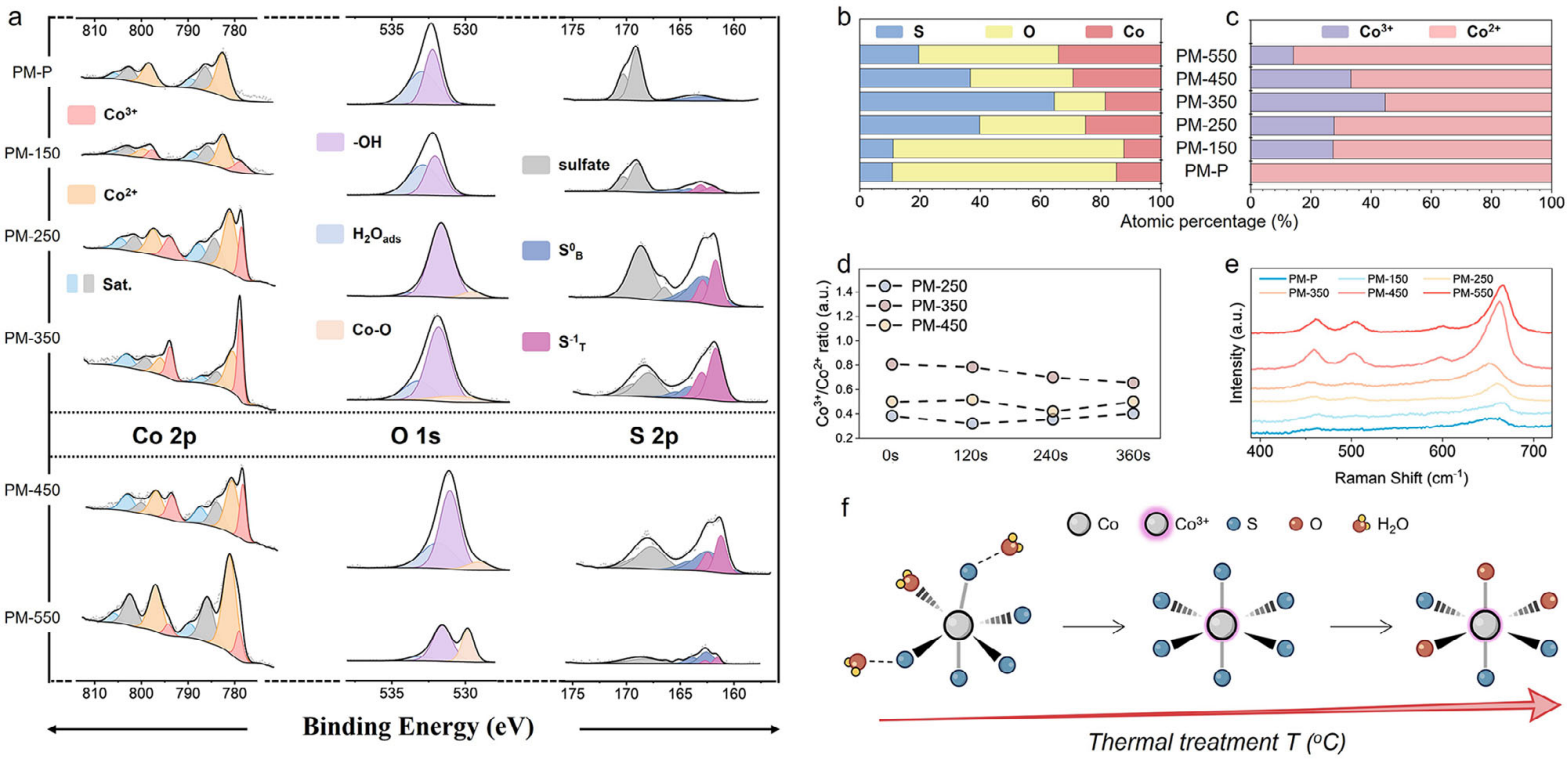
Chemical environment engineering of pre‐catalysts. a) XPS spectra of PM‐P and its annealing derivatives. Atomic percentage of b) S, O, and Co elements and c) Co^2+^ and Co^3+^ deprived from XPS spectra. d) Co^3^+/Co^2^+ ratio calculated from XPS with different Ar+ sputtering times. e) Raman spectra of PM‐P and its annealing derivatives. f) Schematic illustration of the annealing effect on the chemical environment.

Tailoring the microcrystalline structure of PM‐P enables the engineering of a distinct chemical environment. X‐ray photoelectron spectroscopy (XPS) was used to investigate the surface composition and electronic structure of PM‐P and its annealed derivatives (**Figure** **2**a). In the Co 2p spectra of PM‐P, the peaks at 798.4 and 782.6 eV correspond to Co^2^⁺ 2p_1/2_ and Co^2^⁺ 2p_3/2_, respectively,^[^ ^23^, ^38^ ^]^ and these Co^2^⁺ peaks persist across all derivatives. Thermal annealing introduces additional peaks at ≈ 794 and 778.8 eV, attributed to Co^3^⁺ 2p_1/2_ and Co^3^⁺ 2p_3/2_, respectively. Comparative analysis shows that the intensities of Co^2^⁺ and Co^3^⁺ peaks vary with annealing temperature. The Co^3^⁺ to Co^2^⁺ integral area ratio increases with temperature, maximizing at 350 °C with a ratio of 44%, followed by a gradual decline at higher temperatures (Figure 2c; Figures S6–S9, Supporting Information). Thus, PM‐350 exhibits the highest Co^3^⁺ to Co^2^⁺ ratio among all pre‐catalysts.

Thermal treatment altered the chemical structures of oxygen and sulfur in the pre‐catalyst. O 1s XPS spectra reveal three peaks: surface‐adsorbed water (H_2_O) at 533.0 eV, hydroxylated cobalt species (Co─OH) at 532.2 eV, and lattice oxygen (Co─O) at 529.7 eV.^[^ ^39^, ^40^ ^]^ The Co─OH peak shifts to lower binding energy with increasing temperature up to 350 °C, while the Co─O peak appears only above 450 °C, signaling crystalline CoO formation. S 2p spectra show the sulfate peaks at 169 and 167 eV because of the sulfur oxidation, and broad features from 160.1 to 163.8 eV, attributed to bridging sulfur (SB⁰, 163.5 eV) and terminal sulfur (ST^1−^, 162.0 eV),^[^ ^41^ ^]^ respectively. Depth‐profiling XPS analysis after different Ar^+^ sputtering times of the PM‐250, PM‐350, and PM‐450 samples (Figure 2d) revealed a uniform Co^3^⁺/Co^2^⁺ ratio throughout the bulk material across all annealing conditions.

Sulfur enrichment promotes the Co^3^⁺ formation (Figure 2b,c). Thermogravimetric analysis (TGA) (Figure S3, Supporting Information) indicated that the annealing removed surface‐adsorbed H_2_O, exposing excess ST^1−^. Furthermore, annealing promoted the transformation of sulfur from sulfate to its decomposed forms, ST^1−^ and S^2^^−^ species. These modifications in the sulfur environment facilitated the oxidation of cobalt from Co^2^⁺ to Co^3^⁺. Upon reaching the CoO crystallization threshold (≥450 °C), surface hydroxyl groups (─OH adsorbed on cobalt atoms) began to form the CoO lattice, thereby strengthening Co─O bonds. The elevated temperature also facilitated the removal of surface sulfur species, as evident from the diminished S/O ratio observed in Figure 2b. Raman spectroscopy (Figure 2e) further confirmed these changes, displaying four Co_9_S_8_ vibrational modes at 457.1 (E_g_), 501.3 (F_2g2_), 587.4 (F_2g3_), and 651.8 cm^−1^ (A_1g_).^[^ ^40^, ^42^, ^43^ ^]^ Post‐crystallization, these peaks sharpened, with the A1g mode showing a red shift, indicating enhanced long‐range order and tensile strain. The A1g peak of CoO, observed at a higher wavenumber, aligns with the blue shift in PM‐450 and PM‐550.

### 2.3 Distinct Phase Transition During OER

By engineering the chemical structures of pre‐catalysts, distinct phase transitions during OER can be effectively induced. Three distinct pre‐catalysts—PM‐250 (low short‐range order), PM‐350 (highest Co^3^⁺/Co^2^⁺ ratio), and PM‐450 (rigid Co─O bonding)—were chosen to study the in situ phase reconstruction process. Cyclic voltammetry (CV) analysis in 1 M KOH was conducted to assess their OER activities (Figures S10, Supporting Information). All samples displayed a clear redox peak at≈1.05 V in the first cycle, corresponding to the Co^2^⁺/Co^3^⁺ oxidation transition. Further analysis showed higher oxidation states in PM‐350 and PM‐450, with PM‐350 exhibiting a prominent Co^3^⁺→Co⁴⁺ oxidation peak starting at 1.26 V, while PM‐450 showed a fainter signal at 1.34 V.^[^ ^14^, ^27^ ^]^ Additionally, the anodic current intensity in the first CV scan was significantly higher than in subsequent cycles for both catalysts, suggesting irreversible surface reconstruction under OER conditions.

**Figure 3:**
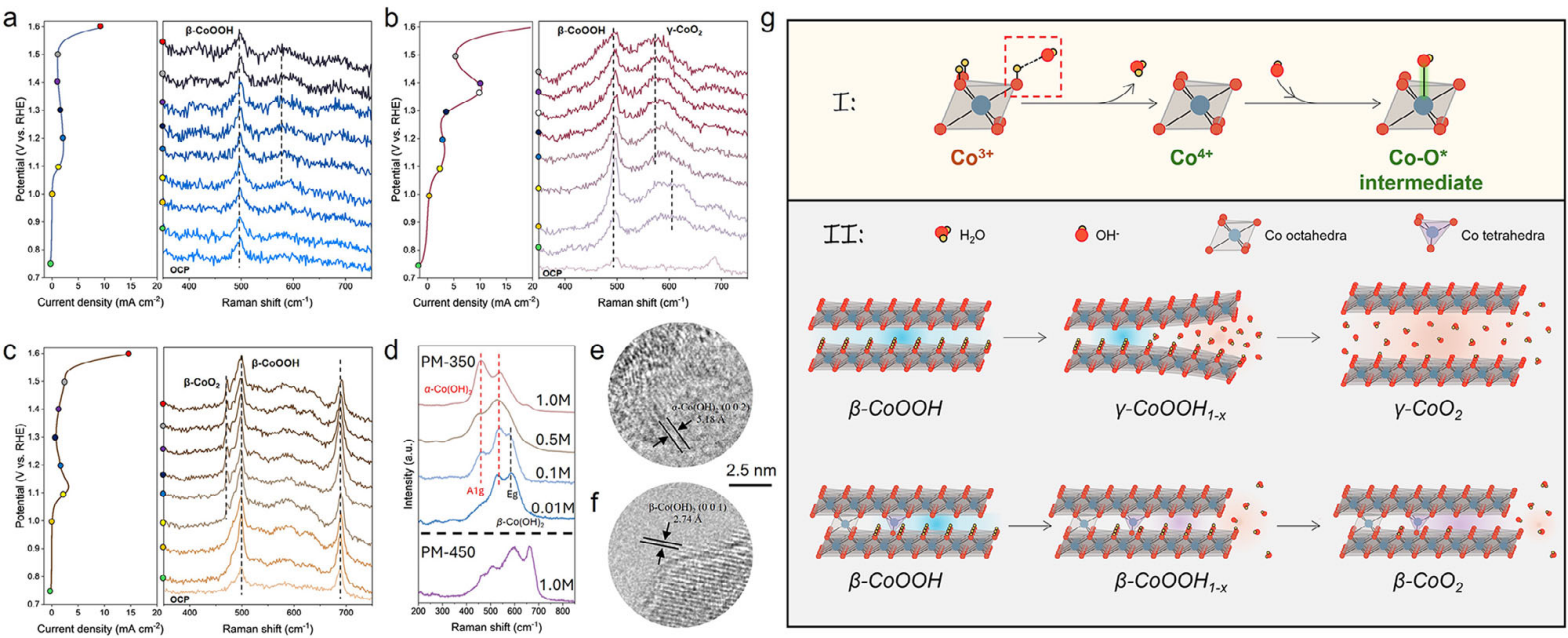
Targeted surface reconstruction during OER. In situ Raman spectra at various potentials of a) PM‐250, b) PM‐350, and c) PM‐450. d) Raman spectra after 30 min OER at 50 mA cm−2 with various KOH concentrations. TEM images after the OER process of e) PM‐350, and f) PM‐450. g) Schematic demonstration of the surface reconstruction process.

In situ Raman spectroscopy was employed to systematically examine phase reconstruction during OER. **Figure** **3**a–c presents half anodic cyclic voltammetry (CV) scans (left panels) alongside corresponding Raman spectra (right panels) at various applied potentials. In alkaline conditions, all pre‐catalysts spontaneously transformed into β‐CoOOH species prior to electrochemical activation, as evidenced by a characteristic Raman vibration mode at 500 cm^−1^ under open‐circuit potential (OCP) conditions.^[^ ^15^, ^39^, ^44^ ^]^ Notably, PM‐350 and PM‐450 displayed an additional Co_3_O_4_‐specific vibrational signature at ≈ 687.5 cm^−1^, the significance of which will be explored in later discussions.^[^ ^45^ ^]^

Additionally, Raman spectra evolved with increasing applied potentials. For PM‐250, the Raman mode at 500 cm^−1^ remained largely unchanged throughout electrochemical testing, with a minor peak emerging ≈ 570 cm^−1^, attributed to the formation of γ‐CoOOH (Figure 3a). For PM‐350, the Raman spectrum displayed characteristic β‐CoOOH peaks, including an A_1g_ vibrational mode of Co─O at 603 cm^−1^ and an E_g_ mode at 500 cm^−1^ (Figure 3b). As the potential increased, the A_1g_ peak intensified, reflecting enhanced structural ordering.^[^ ^39^ ^]^ Simultaneously, the A_1g_ peak position gradually shifted from 603 to 568 cm^−1^, aligning with reported A_1g_ modes of CoO_2_, indicating structural evolution. These changes suggest a transformation from β‐CoOOH to γ‐CoOOH_x_ (where x < 1), a mixed‐valence cobalt oxide containing both Co^3^⁺ and Co⁴⁺.^[^ ^14^, ^15^, ^44^ ^]^

PM‐450 displayed a distinct Raman response compared to PM‐350 (Figure 3c). The A_1g_ peak in PM‐450 showed less enhancement, indicating limited γ‐CoOOH formation. However, a new vibrational mode at 472 cm^−1^ emerged at 1.1 V and above, suggesting an alternative structural reconstruction pathway. Prior studies associate this spectral feature with β‐CoOOH formation, which subsequently evolves into β‐CoO_2_ through further electrochemical oxidation.^[^ ^14^, ^44^ ^]^ The divergent reconstruction pathways likely arise from variations in Co─O bonding rigidity across the samples. In PM‐350, the Co_3_O_4_‐related Raman mode at 687.5 cm^−1^ vanished upon applying potential, indicating complete Co_3_O_4_ dissolution during reconstruction. Conversely, this mode remained prominent in PM‐450 throughout testing, suggesting that the Co_3_O_4_ structure stayed intact and played a dominant role in the reconstruction process under these conditions.

Post‐OER characterization using Raman, XPS, and TEM corroborated the initial hypothesis. Raman spectroscopy was performed on catalysts subjected to 30 min of OER at 50 mA cm^−^^2^ in varying KOH concentrations. As KOH concentration (and hence solution pH) decreased, a portion of α‐Co(OH)_2_ transitioned to β‐Co(OH)_2_ in the PM‐350 sample (Figure 3d).^[^ ^46^, ^47^ ^]^ PM‐450 after OER in 1.0 m KOH showed predominant β‐Co(OH)_2_ phase. The emergence of β‐Co(OH)_2_ phase was a result of restricted interlayer expansion, hindered by the limited intercalation of H_2_O and OH^−^ ions. XPS analysis revealed a decreased sulfur‐to‐oxygen ratio in both post‐OER products, with Co 2p spectra confirming the presence of Co^2^⁺ oxidation states (Figure S11, Supporting Information). TEM imaging provided direct structural evidence, revealing interplanar spacings of 5.18 Å in PM‐350, corresponding to the (002) plane of α‐Co(OH)_2_, and 2.74 Å in PM‐450, matching the (001) plane of β‐Co(OH)_2_ (Figure 3e,f; Figure S12, Supporting Information).

The surface reconstruction process is illustrated in the schematic diagram in Figure 3g. The pre‐catalyst design focuses on enhancing Co^3^⁺ content and exposing sulfide species, which together promote In situ Co⁴⁺ formation during OER. The reconstruction pathway hinges on the presence of rigid Co─O bonds. In systems without such bonds, β‐CoOOH intermediates undergo interlayer expansion via H_2_O and OH^−^ intercalation, leading to γ‐CoO_2_ with increased interlayer spacing.^[^ ^48^ ^]^ Conversely, rigid Co─O bonds hinder this expansion, resulting in β‐CoO_2_ formation.

### 2.4 Mechanistic Insight into the OER Process

**Figure 4:**
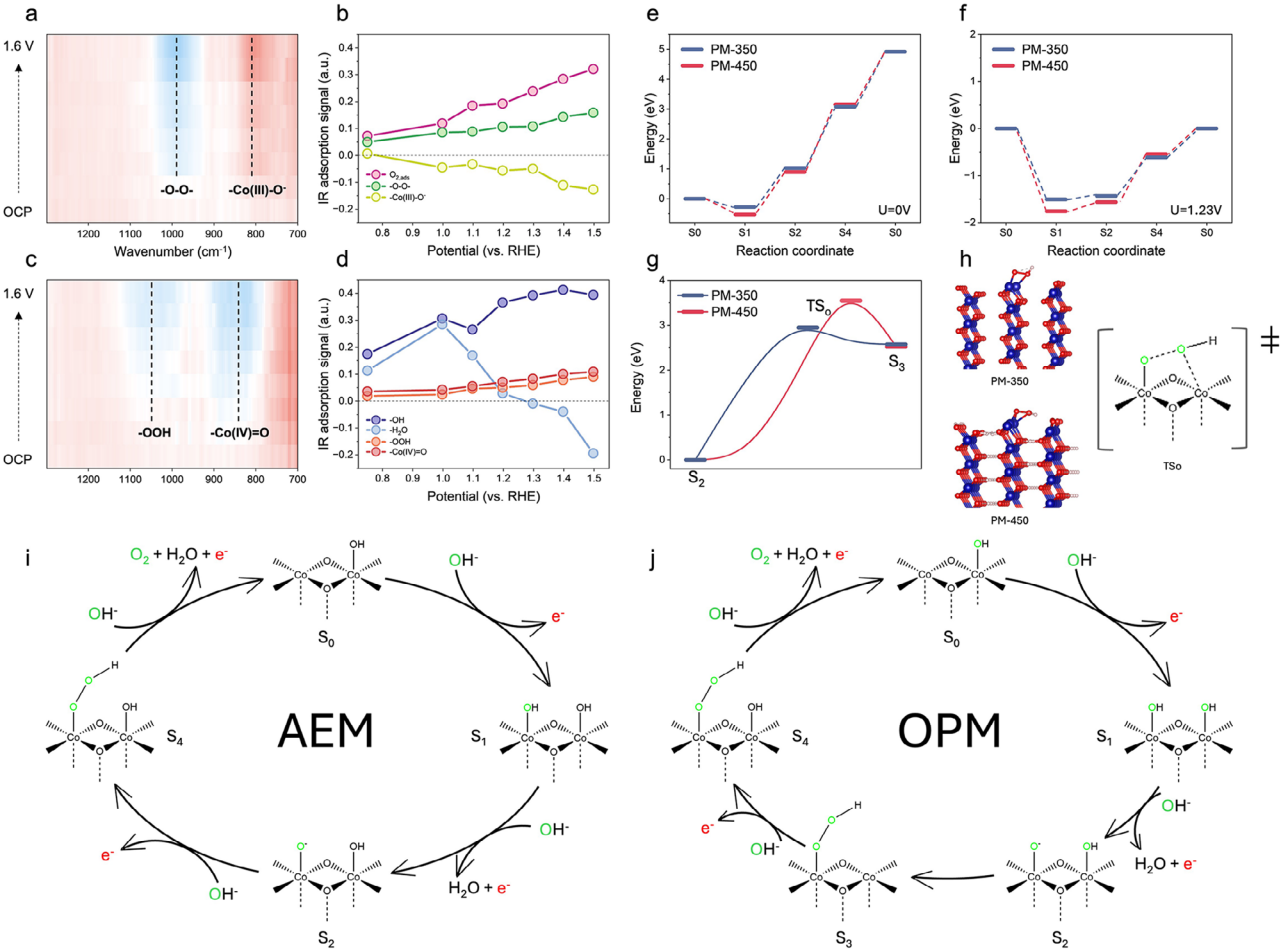
Monitoring the OER mechanism. In situ ATR‐IR measurements of a) PM‐350, and c) PM‐450. Infrared transmittance signals versus potentials of OER intermediates derived from ATR‐IR of b) PM‐350, and d) PM‐450. OER free‐energy diagrams at e) 0 V and f) 1.23 V. g) The kinetic energy barrier of S2→S3 for OPM. h) The configurations of the transition state in the OPM mechanism for PM‐350 and PM‐450. Distinctive types of reaction mechanisms for OER on i) PM‐350, and j) PM‐450.

Increased interlayer spacing in γ‐CoO_2_ enhances OER performance by facilitating greater participation of H_2_O and OH^−^ ions, enabling a more efficient reaction mechanism. In situ attenuated total reflectance infrared (ATR‐IR) spectroscopy was used to probe the mechanistic details of OER.^[^ ^31^, ^49^, ^50^, ^51^, ^52^ ^]^ **Figure** **4**a,c displays the ATR‐IR spectra for PM‐350 and PM‐450. Signals in the 800–900 cm^−1^ range are attributed to oxygen‐containing species without proton involvement, indicating intermediates composed of Co and O.^[^ ^11^, ^12^ ^]^ For PM‐350, the peak intensity at 810 cm^−1^ decreases, while for PM‐450, the peak at 844 cm^−1^ increases.

The vibrational frequency (ν) of a bond is described by the harmonic oscillator model:

(1) $$\nu =\frac{1}{2\pi }\sqrt{\frac{k}{\mu }}$$

where 𝑘 is the force constant (related to bond strength) and 𝜇 is the reduced mass of the vibrating atoms.^[^ ^53^ ^]^ As a result, the 844 cm^−1^ peak could be assigned to ‐Co(IV)═O species, while the 810 cm^−1^ peak, which shifts to a lower wavenumber (indicating reduced vibrational frequency), aligns with a ‐Co(III)‐O^−^ structure characterized by a longer and weaker bond.

Double oxygen‐bonded species, such as superoxide (Co─O─O─Co) and hydroperoxide (Co─O─O─H), typically exhibit vibrational modes in the 980–1100 cm^−1^ range. For PM‐350, a peak at 991 cm^−1^, assigned to the Co─O─O─Co structure, intensifies with increasing applied potential.^[^ ^12^, ^17^ ^]^ Conversely, PM‐450 displays a peak at 1029 cm^−1^, corresponding to Co─O─O─H. The lower wavenumber of the PM‐350 peak compared to PM‐450 results from the higher atomic mass of Co relative to H. According to the reduced mass formula ($\mu =\frac{m1m2}{m1+m2}$), a larger reduced mass‐resulting from heavier atoms like Co‐lowers the vibrational frequency, thereby shifting the peak to a lower wavenumber.^[^ ^54^, ^55^, ^56^ ^]^

For PM‐350, Figure 4b shows the concentration dynamics of intermediates during OER, with a decrease in ─Co(III)─O─ and an increase in ─Co─O─O─Co─. This trend supports the oxygen pathway mechanism (OPM), driven by ‐Co─O─O─Co─ formation,^[^ ^12^, ^57^, ^58^ ^]^ as depicted in Figure 4j. In contrast, PM‐450 follows a conventional AEM, indicated by the steady increase in ─OH, Co─O─O─H, and ─Co^4+^═O with rising potential (Figure 4d). The presence of Co─O─O─H is a key marker of the AEM in PM─450 (Figure 4i). However, the absence of O_2_ detection suggests that this mechanism is slower than the OPM pathway observed in PM‐350.

To verify the In situ FTIR results, we performed first‐principles calculations to elucidate the underlying OER mechanisms. Specifically, we explored two plausible pathways‐AEM and OPM‐to determine the preferred reaction routes for γ‐CoOOH_x_ (x < 1) and β‐CoOOH_y_ (y < 1) catalysts. As previously established, the key distinction between γ‐CoOOH_x_ and β‐CoOOH_y_ arises from the constrained interlayer expansion in the latter, due to limited intercalation of H_2_O and OH^−^ ions. Accordingly, the structural models for β‐CoOOH_y_ and γ‐CoOOH_x_ were constructed to reflect this difference (Figures S14–S16, Supporting Information).

Consistent with prior studies, we considered the Co atoms on the (100) edges as the primary active sites. AEM pathways were modelled on single Co sites, whereas OPM pathways were evaluated over adjacent Co─Co dual sites. For both catalysts, the rate‐determining step (RDS) of the AEM pathway is the formation of *OOH intermediates, corresponding to the transition from state S_2_ to state S_4_ (Figure 4e,f). The calculated free energy barriers for this step are 2.050 eV for γ‐CoOOH_x_ and 2.249 eV for β‐CoOOH_y_ at *U* = 0 V, as shown in Figure 4e. When the applied potential is 1.23 V, we can clearly observe that the overpotentials for γ‐CoOOH_x_ and β‐CoOOH_y_ are 0.82 V and 1.019 V, indicating a faster reaction rate of γ‐CoOOH_x_. The corresponding structures for each OER intermediate of γ‐CoOOH_x_ and β‐CoOOH_y_ are presented in Figures S15 and S16 (Supporting Information).

In contrast, the OPM pathway involves direct coupling of neighboring oxygen species to form the O–O bond. In this mechanism, the S_2_ state with a surface‐bound *O∙ is first formed, followed by O─O bond formation through coupling with a neighboring *OH. As shown in Figure 4g, the kinetic RDS here is the radical coupling step, transitioning from S_2_ to S_3_ via a transition state (TS_o_). This step requires overcoming a kinetic barrier of 2.84 eV for γ‐CoOOH_x_ and 3.46 eV for β‐CoOOH_y_. The lower kinetic energy barrier for γ‐CoOOH_x_ suggests a more favorable OPM route compared to β‐CoOOH_y_.

### 2.5 OER Performance

**Figure 5:**
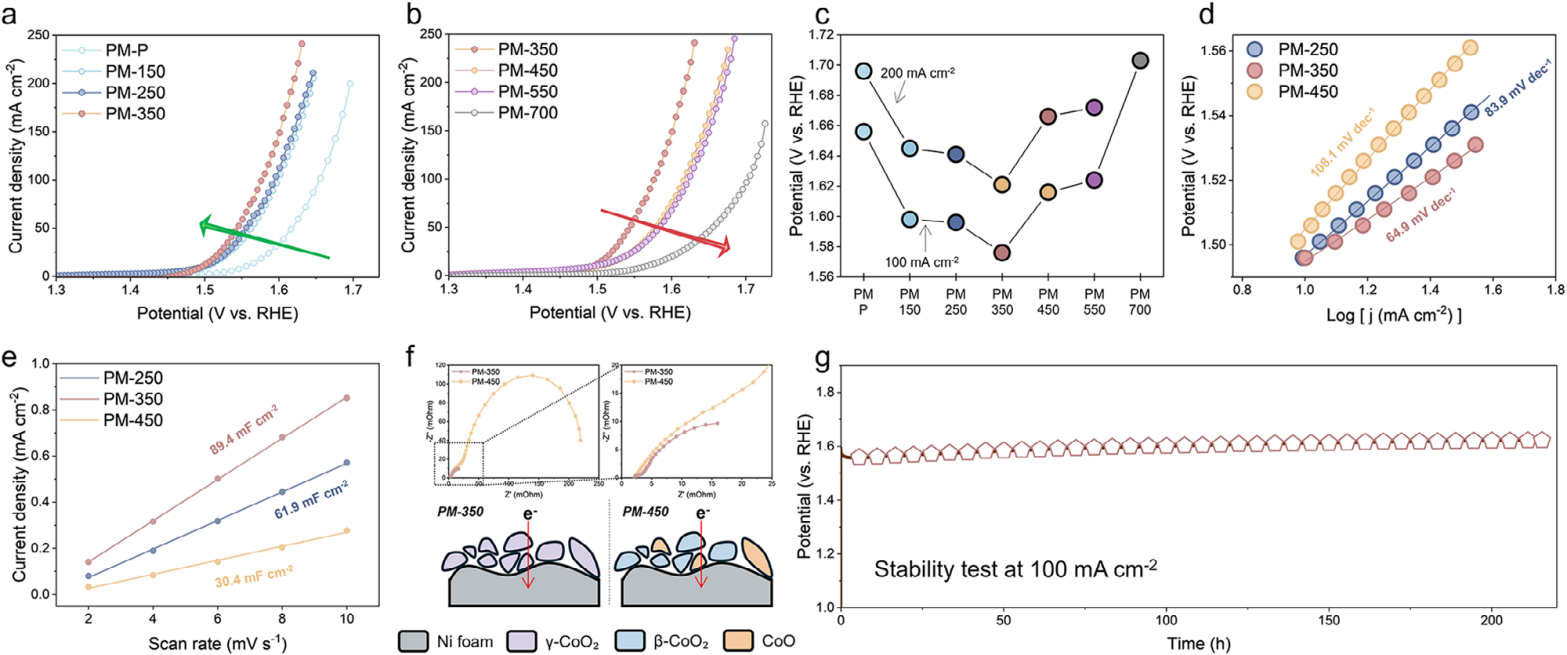
OER performance. LSV curves of samples with a) annealing temperature below 350, and b) annealing temperature above 350. c) Comparison of overpotentials at 100 mA cm^−2^ and 200 mA cm^−2^. d) LSV‐derived Tafel slopes. e) Current density differences versus scan rates to determine ECSA. f) Nyquist plots acquired at 600 mV (vs RHE) in half‐cell. g) Chronopotentiometry curve of PM‐350 at 100 mA cm^−2^.

Deliberately designed pre‐catalysts that favor the OPM pathway significantly enhance OER performance. Catalytic activity was assessed in O_2_‐saturated 1 M KOH using a standard three‐electrode setup. iR‐corrected linear sweep voltammetry (LSV) curves (**Figure** **5**a) revealed that PM‐350 exhibited outstanding OER efficiency, requiring overpotentials of only 354 mV at 100 mA cm^−^^2^ and 391 mV at 200 mA cm^−^^2^. OER activity peaked at an annealing temperature of 350 °C, increasing up to this point and declining at higher temperatures (Figure 5b,c). This volcano‐like trend underscores that γ‐CoO_2_ outperforms β‐CoO_2_, and the OPM mechanism provides a substantially faster reaction pathway compared to the conventional AEM.

To evaluate the intrinsic catalytic activity, Tafel slopes were calculated from LSV curves (Figure 5d). PM‐350 exhibited a lower Tafel slope of 64.9 mV dec^−1^ compared to PM‐250 (83.9 mV dec^−1^, no Co⁴⁺) and PM‐450 (108.1 mV dec^−1^, β‐CoO_2_ phase), highlighting its superior OER kinetics. The electrochemically active surface area (ECSA) was assessed via double‐layer capacitance (Cdl) in the non‐faradaic potential region. PM‐350 shows a Cdl of 89.4 mF cm^−^^2^, surpassing PM‐250 (61.9 mF cm^−^^2^) and PM‐450 (30.4 mF cm^−^^2^) (Figures 5e; Figure S17, Supporting Information).

Electrochemical impedance spectroscopy (EIS) was employed to further investigate the charge transfer resistance (R_ct_) during OER. The Nyquist plots revealed distinct differences between the samples: PM‐450 exhibited two semicircles, whereas PM‐350 displayed only one, suggesting the presence of phase separation in PM‐450. A detailed analysis of the first semi‐circle region showed that PM‐350 possesses a significantly lower R_ct_ compared to PM‐450. This observation highlights the superior charge transfer kinetics of γ‐CoO_2_ relative to its β‐CoO_2_ counterpart. The alignment of EIS, ECSA, and the volcano‐shaped OER activity in LSV curves underscores the critical role of phase‐dependent properties (γ‐CoO_2_ versus β‐CoO_2_) and the OPM pathway in optimizing catalytic performance.

Long‐term stability is a key metric for assessing the catalytic performance. PM‐350 maintains stable catalytic activity with no significant degradation over 200 h at a constant current density of 100 and 500 mA cm^−^^2^, demonstrating its excellent durability (Figure 5g; Figure S18, Supporting Information). The initial voltage surge, followed by stabilization, is attributed to the In situ formation of γ‐CoO_2_ during the OER.

## 3 Conclusion

In summary, we propose a design strategy focused on enhancing Co^3^⁺ species in the pre‐catalyst to facilitate the in situ formation of γ‐CoO_2_, which accelerates OER via an OPM mechanism. Incorporating rigid Co–O bonds alters the reconstruction pathway, favoring β‐CoO_2_ formation with reduced interplanar spacing, which follows the conventional AEM. This approach was achieved using polymorphous cobalt oxysulfide and its annealed derivatives. The in situ Raman spectroscopy and ex situ characterizations confirm the roles of Co^3^⁺ and Co─O bonding in structural evolution. In situ ATR‐IR spectroscopy further clarified the distinct reaction pathways of each active phase. The pre‐catalyst optimization promotes the γ‐CoO_2_ formation, leading to superior OER performance, which highlights the effectiveness of this design strategy.

## Conflict of Interest

The authors declare no conflict of interest.

## Supporting information



Supporting Information

## Data Availability

The data that support the findings of this study are available in the supplementary material of this article.
