# Supplementary material for: Identifying Beneficial and Adverse Co4+ Species in Cobalt‐Based Oxygen Evolution Catalysts via Precursor Polymorphism Engineering
Source: Adv Sci (Weinh). 2025 Sep 29;12(43):e09145. doi: 10.1002/advs.202509145 (PMC12631884; doi:10.1002/advs.202509145)
Supplement: Supplementary file 1 — Supporting Information [file ADVS-12-e09145-s001.docx]

Supporting Information

**Identifying Beneficial and Adverse Co⁴⁺ Species in Cobalt-Based Oxygen Evolution Catalysts via Precursor Polymorphism Engineering**

*Zhongheng Li, Zheng Shu, Lun Li, Wendi Zhang, Jiaqian Kang, Chengcheng Zhong, Ziwen Feng, Jinxian Feng*, Hou Ian*, Hui Pan**

Institute of Applied Physics and Materials Engineering, University of Macau Taipa, Macau SAR 999078, China. Emails: [jinxianfeng@um.edu.mo](mailto:jinxianfeng@um.edu.mo); houian@um.edu.mo; [huipan@um.edu.mo](mailto:huipan@um.edu.mo)

^*^ Corresponding authors

**Experimental Procedures：**

**Materials:** CoSO_4_ · 7H_2_O, 2-methylimidazole, Nafion solution (5 wt %) and thioacetamide (TAA) were obtained from Sigma-Aldrich. All the chemicals are reagent-grade and used as received.

**Synthesis of Polymorphous Cobalt Oxysulfide:** The synthesis of Co-MOF was based on a previous procedure with modifications ^[1-3]^. Typically, 3 mmol CoSO_4_ · 7H_2_O was dissolved in 25 mL of methanol to form a solution, which was subsequently poured into 25 mL of methanol containing 24 mmol 2-methylimidazole. After thorough mixing, the solution was incubated at room temperature for 24 h. The resulting purple precipitates were collected by centrifuging, washed with water and methanol in sequence for at least three times, and finally dried in vacuum at 50 °C overnight. Then, 80 mg of Co-MOF was redispersed into 40 mL of ethanol, followed by the addition of 0.12 g of TAA. Thereafter, the mixture was transferred into a Teflon-lined stainless-steel autoclave and heated at 120 °C for 4 h. The formed cobalt oxysulfide precursors were centrifuged and washed with ethanol for several times. Finally, samples were annealed in a tube furnace under a continuous argon flow (45 sccm) at temperatures of 150, 250, 350, 450, 550, and 700 °C for 2 hours each. The temperature increase rate was set to be 4 ^o^C/min. After each 2-hour heating period, the samples were allowed to cool naturally to room temperature within the furnace under the maintained argon atmosphere.

**Characterization:** X-ray diffraction (XRD) was conducted using a Rigaku Smart lab X-ray diffractometer with Cu Ka radiation (λ = 0.15 418 nm). Morphological analysis was performed via field emission scanning electron microscopy (FE-SEM) using Zeiss Sigma instrument at the acceleration voltages of 3 kV. High-resolution transmission electron microscopy (HRTEM) and energy-dispersive X-ray spectra analysis (EDX) were carried out on a Talos F200S system at an acceleration voltage of 200 kV. X-ray photoelectron spectroscopy (XPS) measurements were obtained using a Thermo Fisher Scientific instrument equipped with Al Ka X-ray (*hν* = 1486.7 eV) as the excitation source and C1s (284.8 eV) for calibration. Raman spectroscopy was conducted using a Micro Raman System (Horiba LABHRev-UV) with an excitation laser wavelength of 532 nm. For in situ Raman measurement, a flow cell with three electrodes was employed in conjunction with a Chenhua electrochemical workstation. By gradually varying the applied potential to the electrode, the Raman spectra of the samples were acquired. In-situ Fourier transform infrared absorption spectrometer (FTIR) were performed on a Shimadzu IRXross FTIR. Specifically, an electrochemical workstation (CHI 660E) was connected to the insitu FTIR test setup (external reflection mode). Starting from an overpotential of 0.75 V vs. RHE, a voltage change gradient of 100 mV was applied, and activation occurred at the measured voltage for 1 minute before the test. Unlike the in-situ Raman test, the in-situ FTIR test includes a background scan for each measurement.

**Preparation of Working Electrodes:** To make a working electrode for LSV and stability test. 8 mg of catalyst, 1 mg of conductive carbon black (ECP600JD, Ketjenblack®) was homogenously mixed with 250 μL of DI water, 250 μL of ethanol and 50 μL of 5 *wt*% Nafion and sonicated to make a homogeneous ink. Then 50 μL of catalyst ink was dropped onto Ni foam, dried at room temperature and used for electrochemical measurements. For CV tests and all the in-situ tests, carbon paper was selected as the substrate. Besides to ensure the fully reconstruction during the tests, only 20 μL of catalyst ink was dropped onto carbon paper.

**Electrocatalytic OER:** All the electrochemical tests were conducted using a three-electrode configuration on ModuLab XM electrochemical workstation in 1 M KOH (PH = 14) at room temperature. The working, reference, and counter electrodes were PM-P and its derivatives, Hg/HgO (1 M KOH) and graphite rod, respectively. The samples underwent activation through 50 cycles of cyclic voltammetry (CV) at a scan rate of 50 mV s^−1^ under the potential window of 0–0.6 V versus Hg/HgO for the stable test. The polarization curves were recorded via linear sweep voltammetry (LSV) at a scan rate of 5 mV s^−1^ with 85% iR correction. The double-layer capacitance was measured at different scan rates (2, 4, 6, 8, 10 mV s^−1^) within the potential window of 0.3–0.4 V versus RHE in 1 M KOH. EIS measurements were performed under 0 V (vs RHE) across a frequency range of 0.01–10^5^ Hz. Stability test was carried out at 100 mA cm^−2^ for 200 h. The potential was calibrated to the reversible hydrogen electrode (RHE) using the equation E_RHE_ = E_Hg/HgO_ + 0.059 × PH +0.098. The overpotential (η) was determined using the following equation:

η=E_RHE_ – 1.23 V

**Computational Methods**

The spin-polarized DFT calculations were performed using the projector-augmented wave (PAW) pseudo-potentials in the Vienna *ab initio* Simulation Package (VASP 5.4.1).^[4, 5]^ The Perdew–Burke–Ernzerhof (PBE) form of the generalized gradient approximation (GGA) was used to depict the exchange-correlation functional with a plane-wave cutoff energy of 520 eV.^[6, 7]^ The structures of CoOOH and CoOO used for OER process were modelled by a 3 ×1 supercell slab of (100) edge containing 4 layers.^[8]^ To avoid the periodic interaction, a ~15 Å vacuum layer was adopted in the *z*-axis, and the van der Waals dispersion correction was considered via the DFT-D3 method.^[9]^ For 3*d* Co atoms, the Hubbard *U*_eff_ correction with a value of 3.32 that is consistent with the value of Materials Project was included to describe the localized *d* electrons. All structures were fully optimized with a 2 × 2 × 1 *k*-point mesh within Monkhorst-Pack scheme (the bottom three layers are fixed) by the conjugate-gradient (CG) algorithm until the total energy and Hellmann-Feynman force are less than 1 × 10^-5^ eV and 0.03 eV Å^–1^, respectively. According to the computational hydrogen electrode (CHE) model proposed by Nørskov *et al.*,^[10, 11]^ the Gibbs free energy change for OER can be calculated as:

$\Delta G=\Delta E+\Delta ZPE-T\Delta S$ (1)

where ∆*E* represents the energy difference of the system before and after elementary reaction, ∆*ZPE* and ∆*S* are the contributions of zero-point energy and entropy change, which can be calculated from vibrational frequencies, and *T* is the temperature which is set to 298.15 K in our work. The calculations of *ZPE* and *TS* are based on the following equations:

$ZPE=\frac{1}{2}\sum_{i} h\nu_{i}$ (2)

$-TS=k_{B}T\sum_{i} \ln\left( 1-e^{-\frac{h\nu_{i}}{k_{B}T}} \right)-\sum_{i} h\nu_{i}\left( \frac{1}{e^{\frac{h\nu_{i}}{k_{B}T}}-1} \right)$ (3)

where *k*_B_, h and *v*_i_ denote Boltzmann constant, Planck constant and vibrational frequencies of mode *i*, respectively. As for OER in an alkaline electrolyte, four elementary proton-coupled electron transfer (PCET) steps can be proceeded as follows:

$OH^{-}+*\to OH^{*}+e^{-}$ (4)

$OH^{*}+OH^{-}\to O^{*}+H_{2}O\left( l \right)+e^{-}$ (5)

$O^{*}+OH^{-}\to OOH^{*}+e^{-}$ (6)

$\mathrm{OO}H^{*}+OH^{-}\to*+O_{2}(g)+H_{2}O\left( l \right)+e^{-}$ (7)

Therefore, the reaction Gibbs free energy of equations (7)-(10) for OER can be calculated using the following equations:

$\Delta G_{1}=G_{OH*}+{1/2G}_{H2}-G_{*}-G_{H2O}$ (8)

$\Delta G_{2}=G_{O*}+{1/2G}_{H2}-G_{OH*}$ (9)

$\Delta G_{3}=G_{OOH*}+{1/2G}_{H2}-G_{O*}-G_{H2O}$ (10)

$\Delta G_{4}=G_{*}+G_{O2}+{1/2G}_{H2}-G_{OOH*}$ (11)

The Gibbs free energy of the O_2_ molecule should be determined according to *G*_O2_(g) = 2*G*_H2O_(l) − 2*G*_H2_ + 4 × 1.23 (eV) due to the poor description of O_2_ molecule high-spin ground state in DFT calculations. For OER, the onset potential can be calculated by

$U_{\mathrm{OER}}^{\mathrm{onset}}=-min\{\Delta G_{1},\Delta G_{2},\Delta G_{3},\Delta G_{4}\}$ (12)

The overpotential can be calculated using

$\eta_{\mathrm{OER}}=\max\left\{ \Delta G_{1},\Delta G_{2},\Delta G_{3},\Delta G_{4} \right\}/e-1.23V$ (13)

To perform the transition state calculations in OPM mechanism, the climbed nudged elastic band (CI-NEB) method was performed by the quick-min algorithm with 3 insert images between initial and final states.^[12]^ All geometric structures were visualized using the VESTA package.^[13]^

**
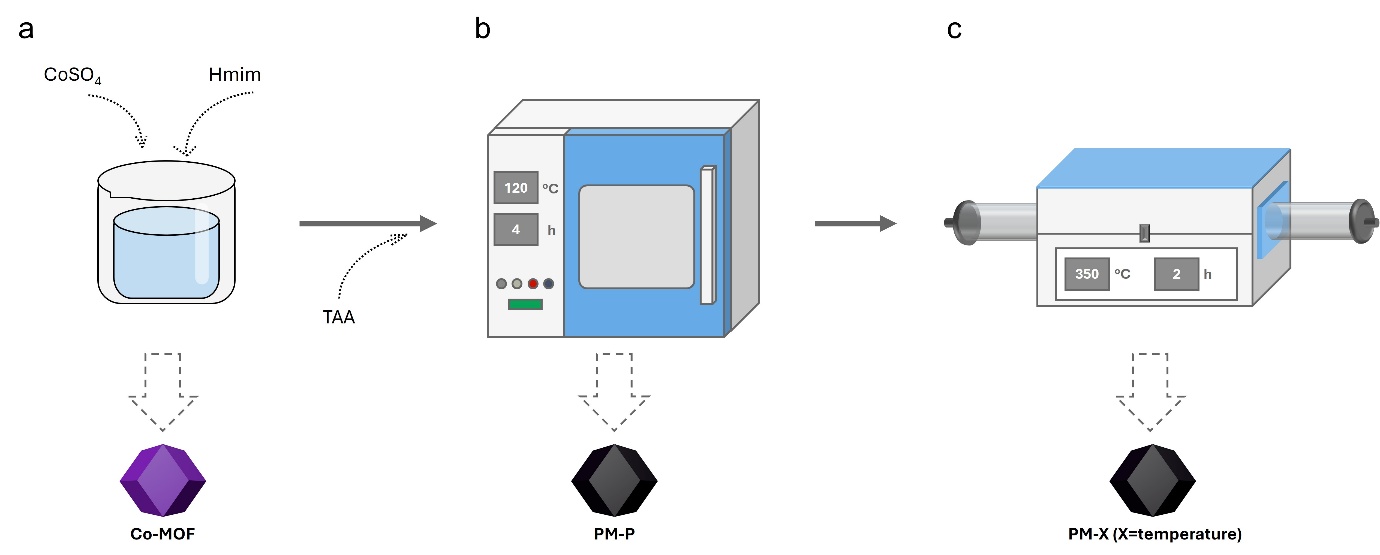
**

**Figure S1.** The synthesis process of cobalt oxysulfide pre-catalysts. (a) Synthesis of ZIF-67, (b) sulphur exchange, and (c) annealing.

**
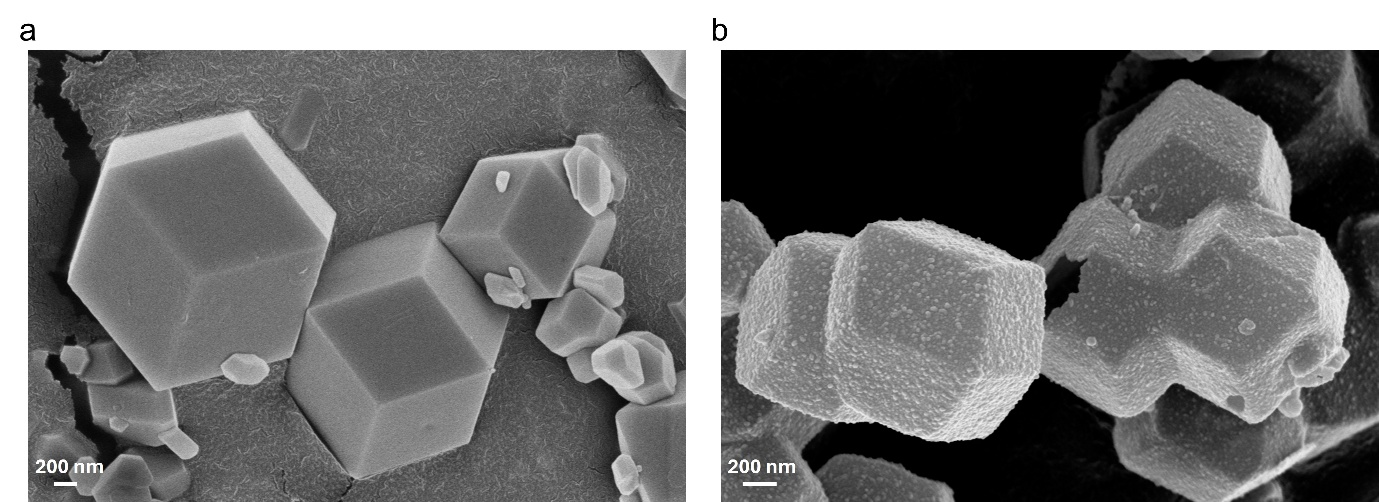
**

**Figure S2.** The SEM images of Co-MOF and cobalt oxysulfide precursor. (a) Co-MOF. (b) PM-P.

Initially, S²⁻ ions from TAA rapidly react with metal ions on the MOF surface, forming a thin sulfide shell. Further sulfidation requires ion diffusion through the shell. The smaller metal ions (74 pm) diffuse outward more readily than S²⁻ ions (184 pm) diffuse inward, dissolving the inner MOF. Outward-diffusing metal ions react with S²⁻ at the outer surface, creating a hollow interior. The MOF thus self-templates into hollow polyhedra.

**
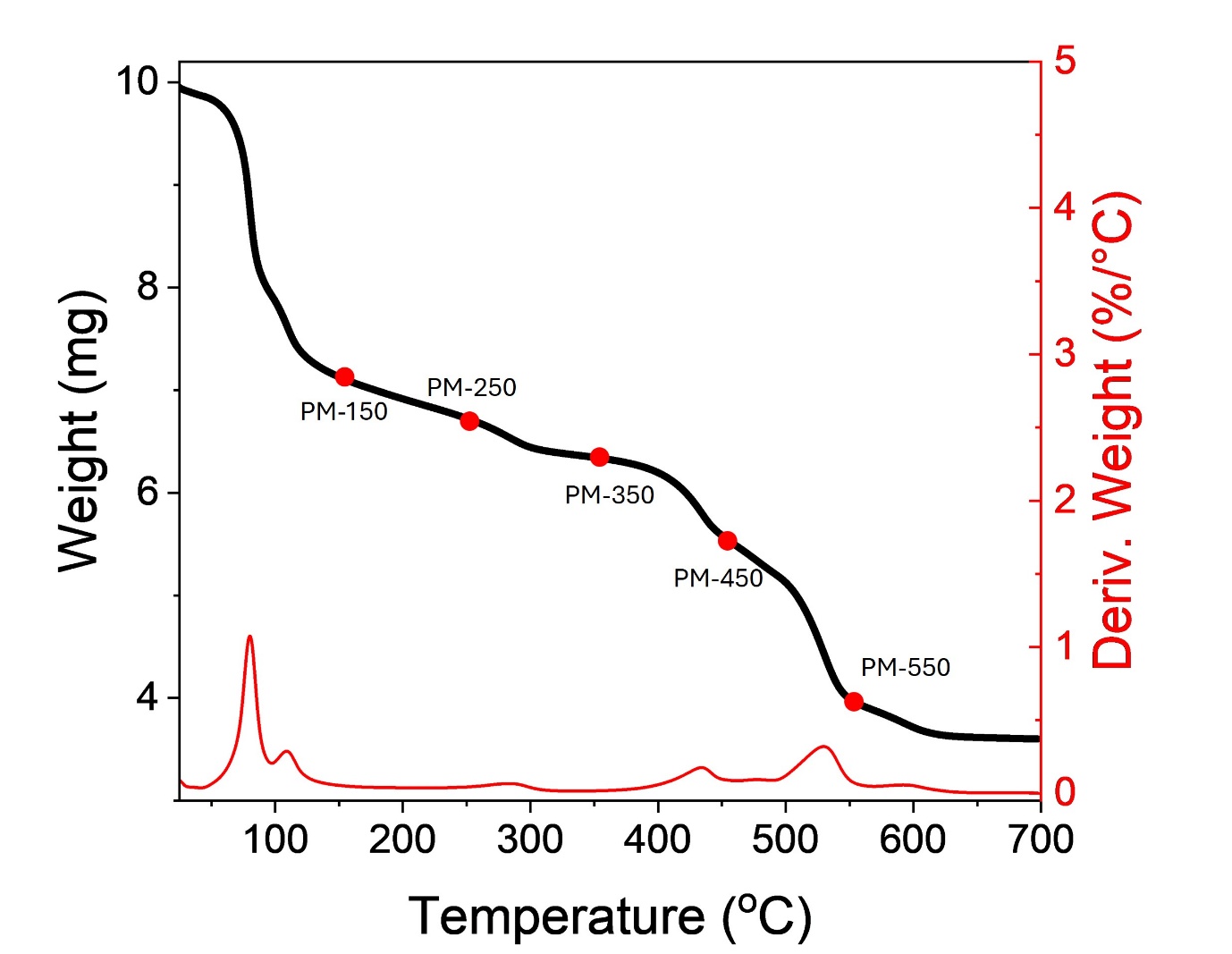
**

**Figure S3.** TGA test of cobalt oxysulfide.

**
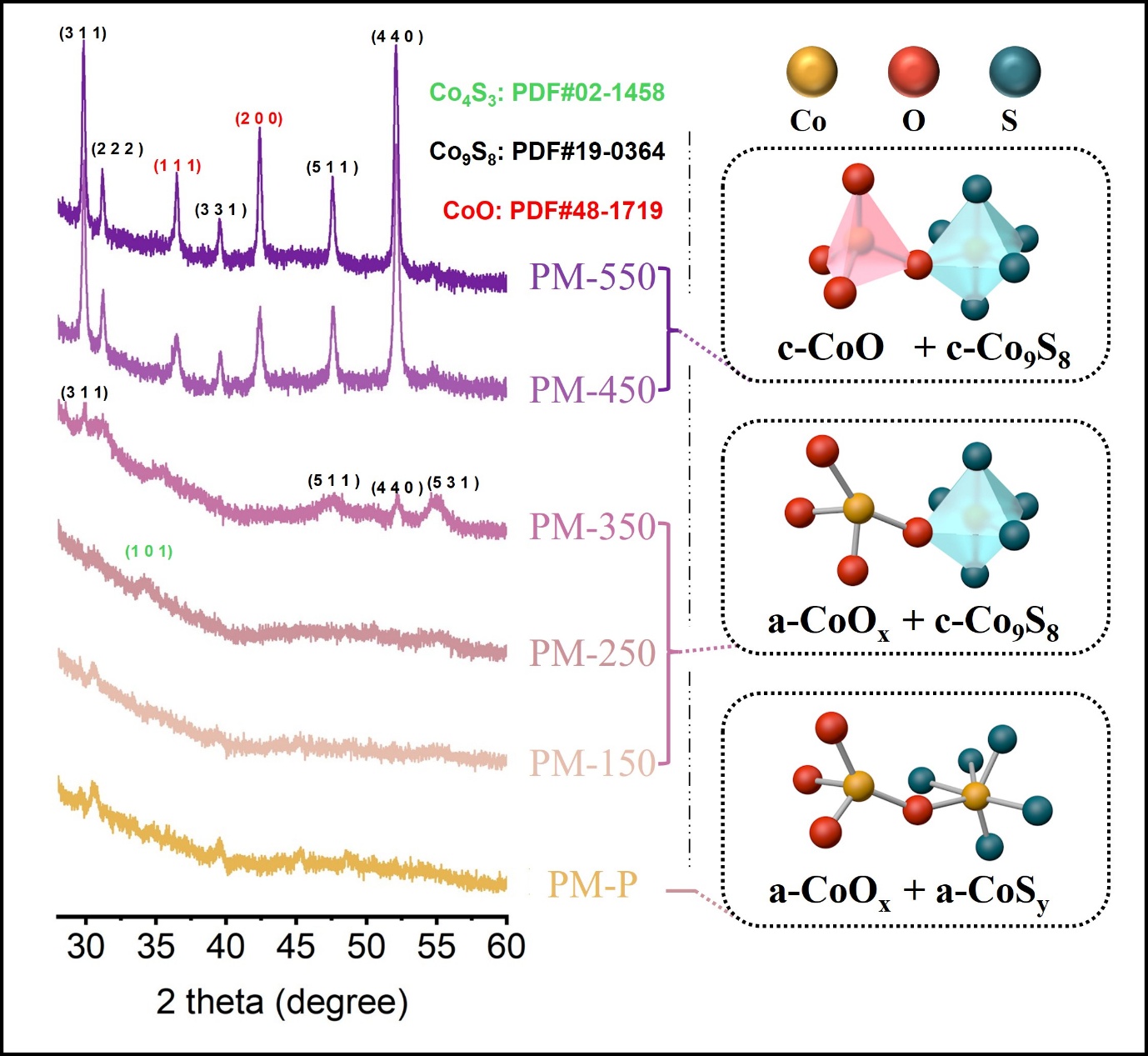
**

**Figure S4.** The XRD of cobalt oxysulfide precursor (PM-P) and its derivatives.

From this figure, we can clearly observe the gradual crystallization of PM-P under annealing condition. The cobalt sulfide phase is the first to crystalize as evidenced by the emergence of (3 1 1), (5 1 1), (4 4 0) and (5 3 1) peaks. Then the cobalt oxide phase began to crystalize after 450 ^o^C as evidenced by (1 1 1) and (2 0 0) peaks.


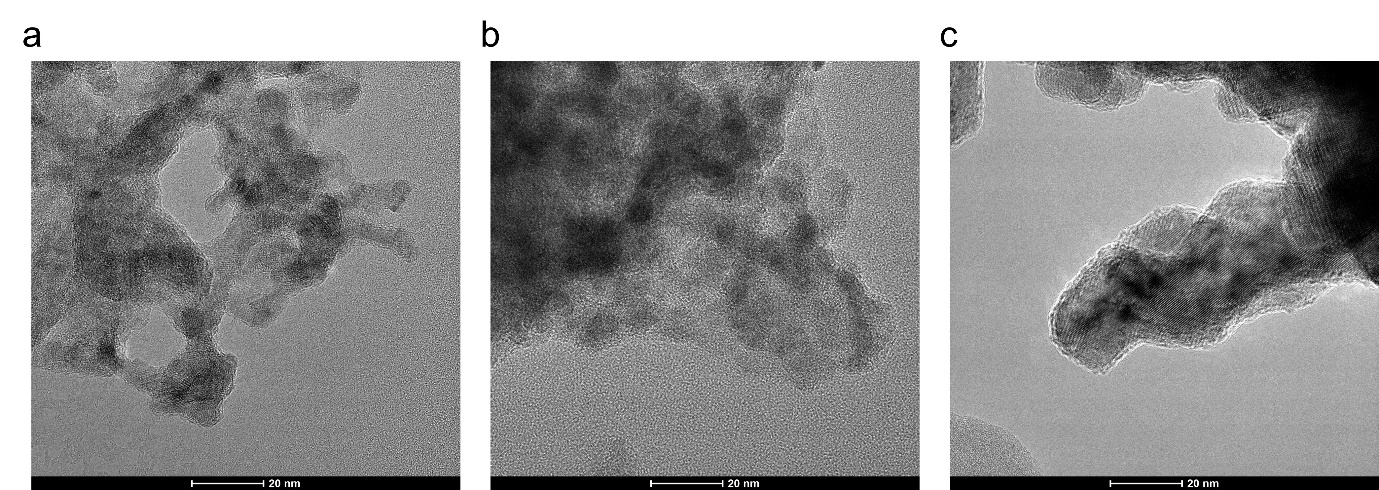


**Figure S5.** TEM image of (a) PM-250, (b) PM-350, (c) PM-450.

A polycrystalline material consists of many small crystals called grains. Each grain is a region where the atomic arrangement is orderly, but at the grain boundaries, where grains meet, this order is disrupted. When a material is annealed, it’s heated to a temperature where new, strain-free grains can form and grow. Higher temperatures boost atomic mobility, as the annealing temperature increases, atoms gain more energy, allowing them to diffuse more readily. This enhanced mobility enables grain boundaries to move, letting larger grains expand by consuming smaller ones.

PM-450 exhibits the largest grain size, while PM-250 has the smallest. This indicates that new grains form and expand once the annealing temperature surpasses critical thresholds.


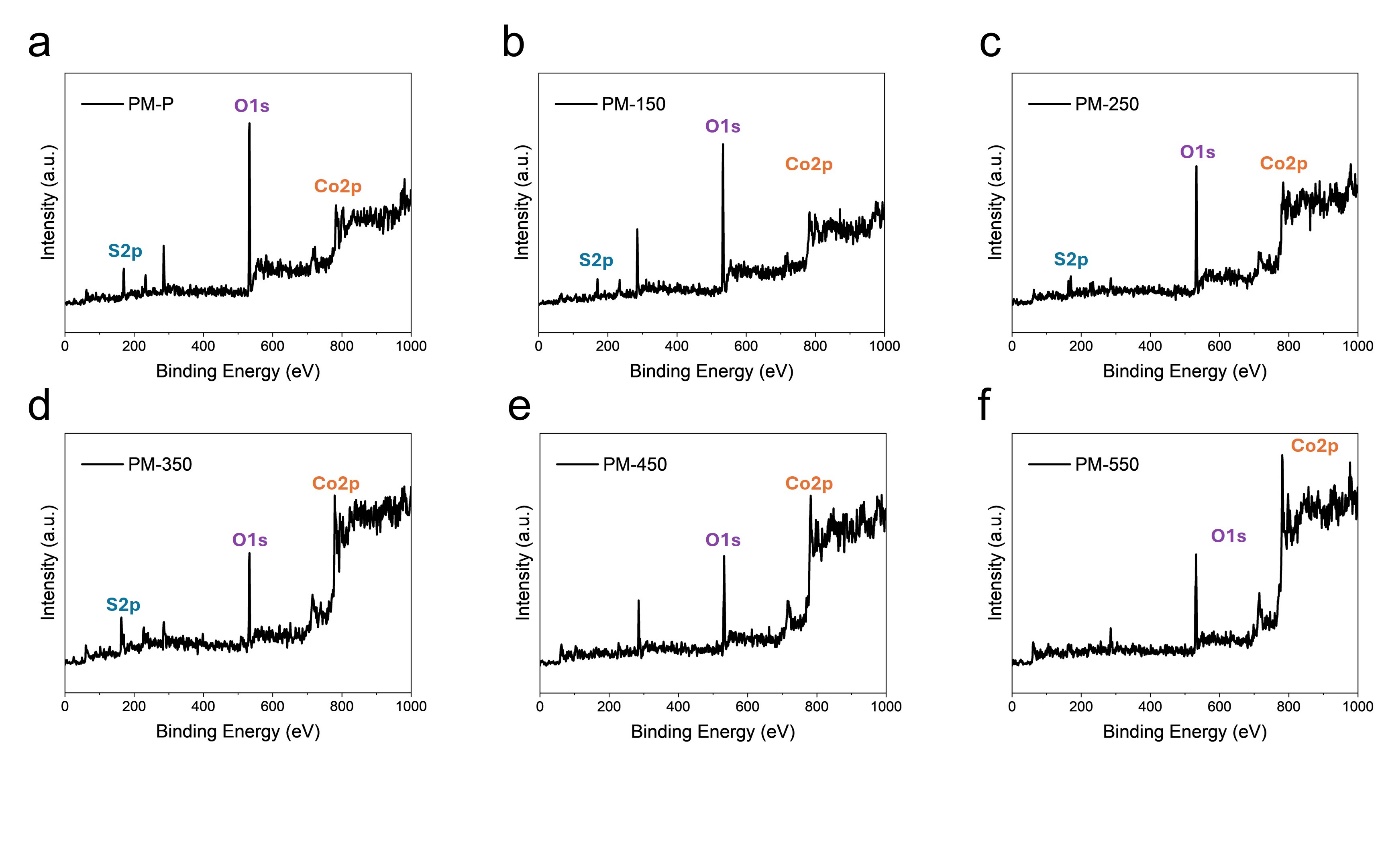


**Figure S6.** XPS full spectra of (a) PM-P, (b) PM-150, (c) PM-250, (d) PM-350 (e) PM-450, (f) PM-550.


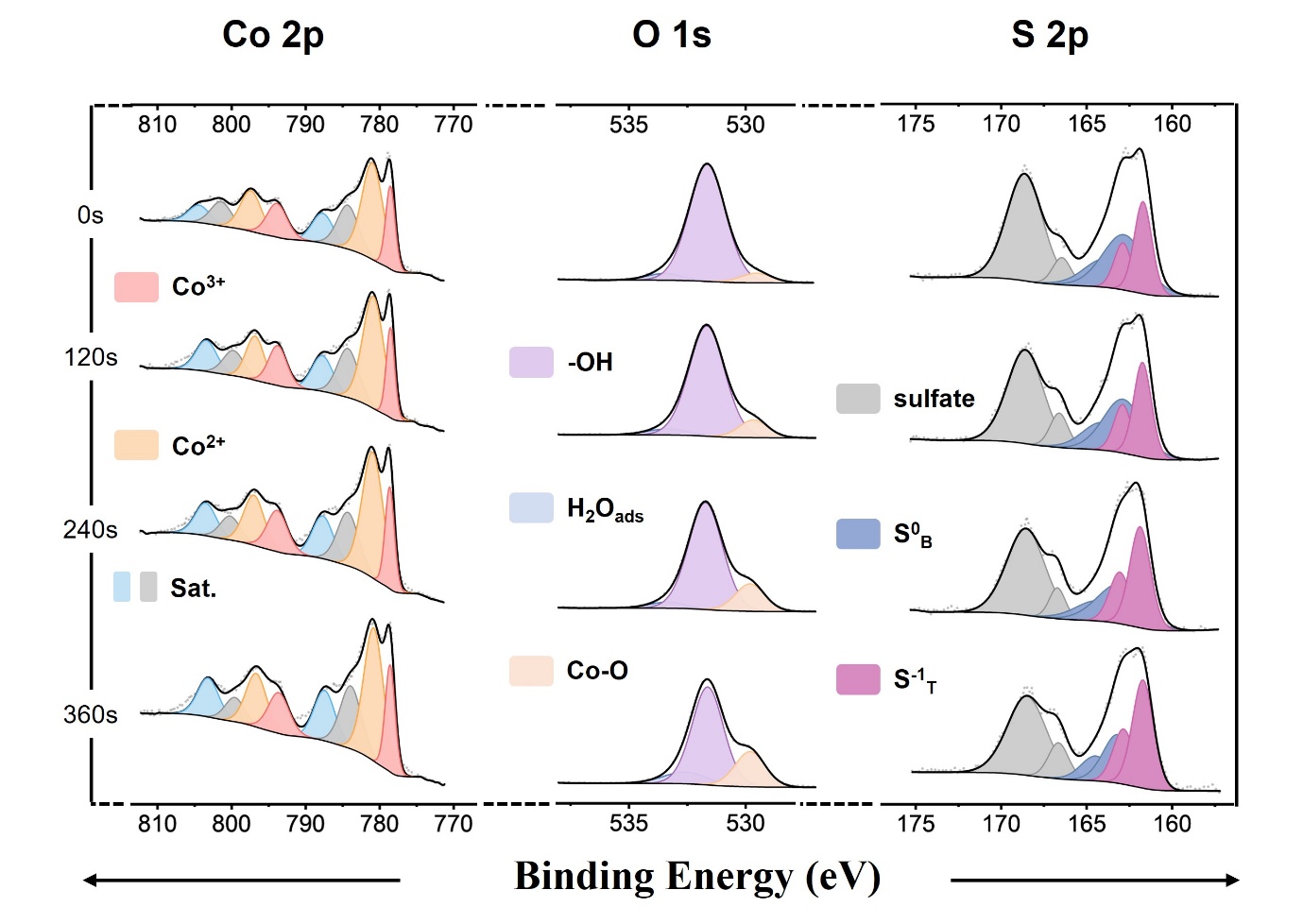


**Figure S7.** Depth-profiling XPS analysis of PM-250.


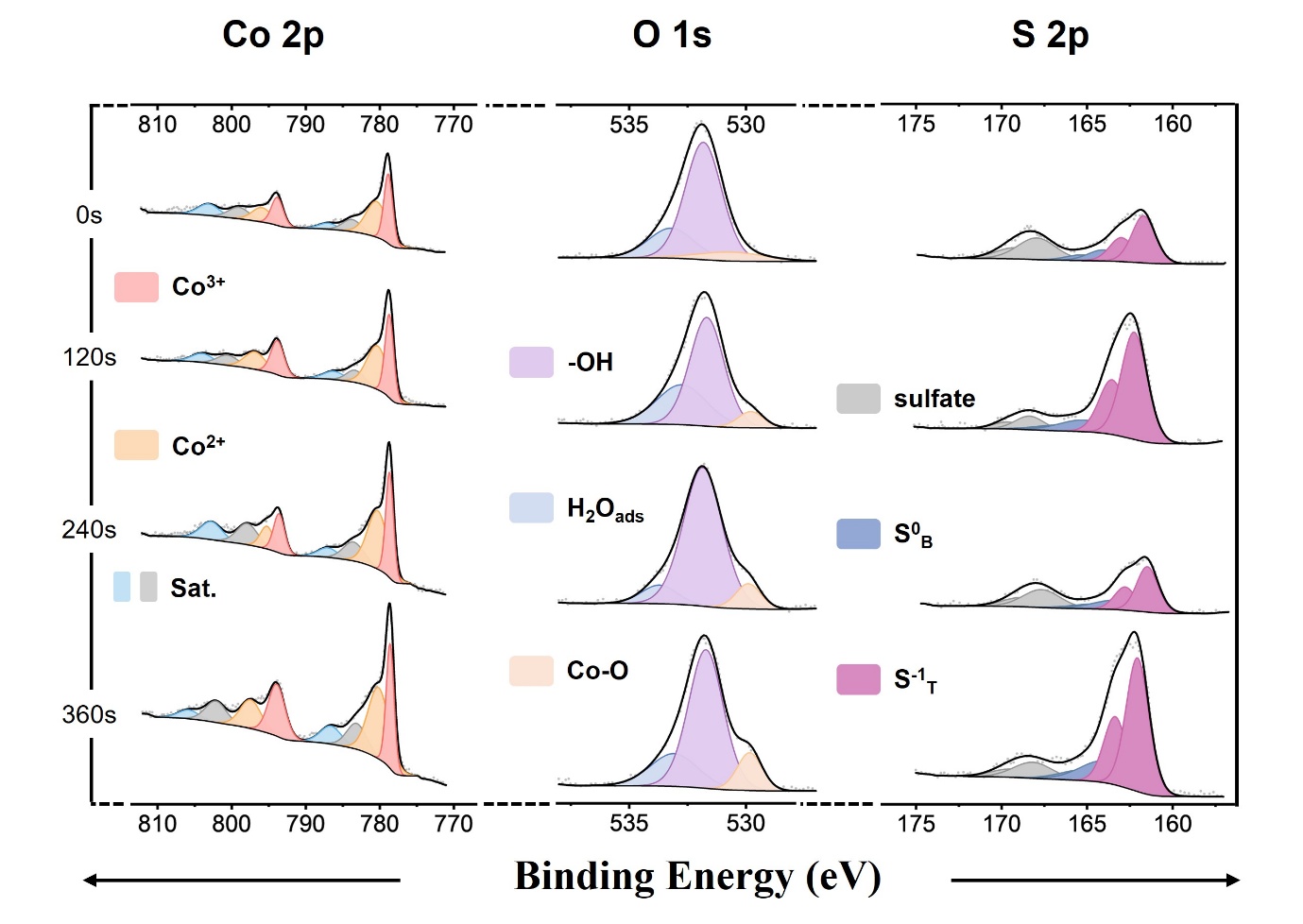


**Figure S8.** Depth-profiling XPS analysis of PM-350.


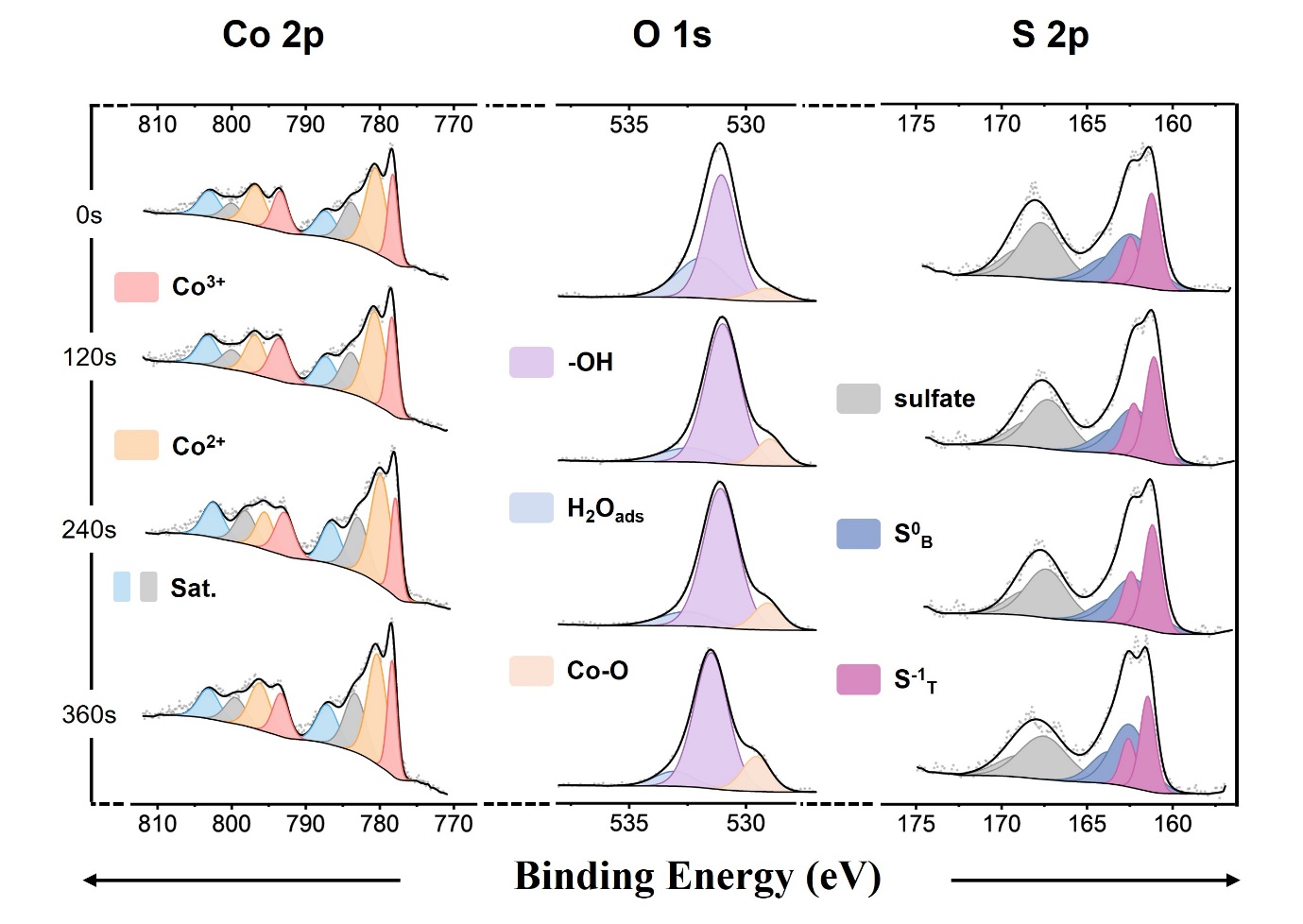


**Figure S9.** Depth-profiling XPS analysis of PM-450.


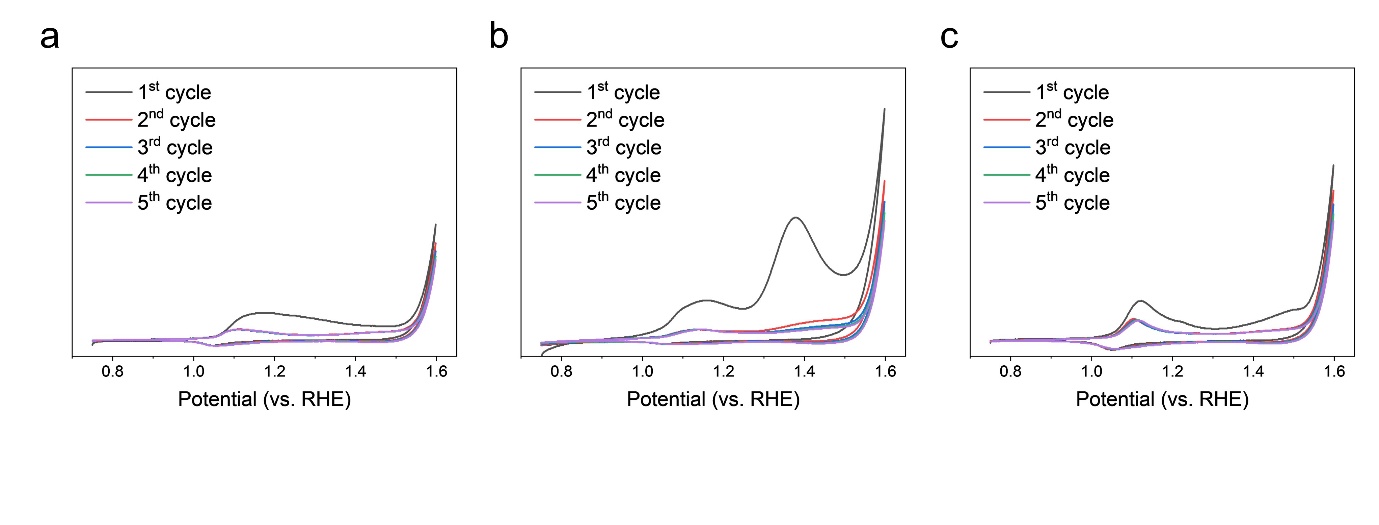


**Figure S10.** CV curves of (a) PM-250, (b) PM-350, and (c) PM-450 tested under the scan rate of 10 mV cm^-2^. The setup was a common in-situ Raman setup with a 3-electrode system. The substrate for this CV test is carbon paper to avoid the influence of Ni’s redox peaks.

For CV tests, the first red-ox peak pair can be assigned to the Co^2+^/Co^3+^ redox couple, while the second red-ox peak pair, occurring at higher potentials, can be attributed to the Co^3+^/Co^4+^ transition. The presence of this second pair will serve as strong electrochemical evidence for the formation of Co^4+^ during the reaction.


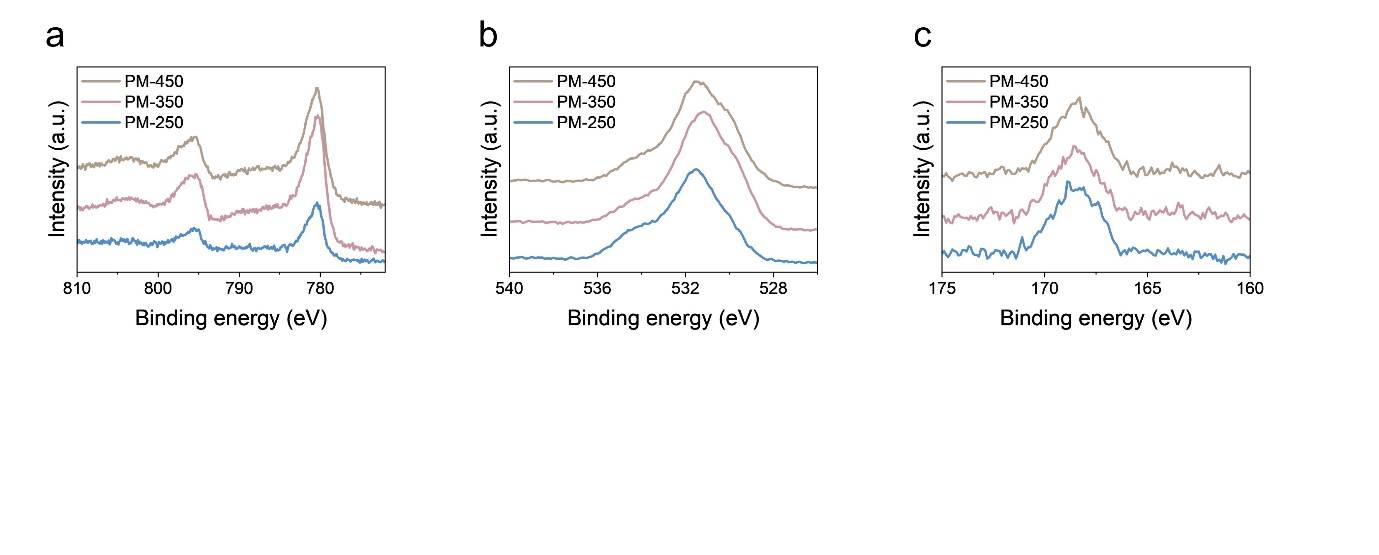


**Figure S11.** XPS spectra of the post-OER samples (10 mA cm^-2^ for 10 min). (a) Co_2p_ spectra, (b) O_1s_ spectra, and, (c) S_2p_ spectra.


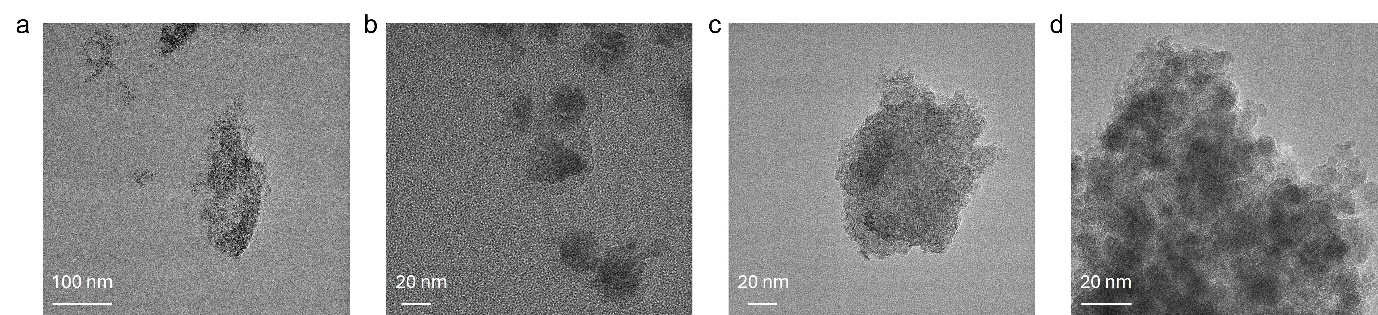


**Figure S12.** TEM image of post-OER samples (10 mA cm^-2^ for 10 min). (a) PM-250, (b) PM-250, (c) PM-350, and (d) PM-450.


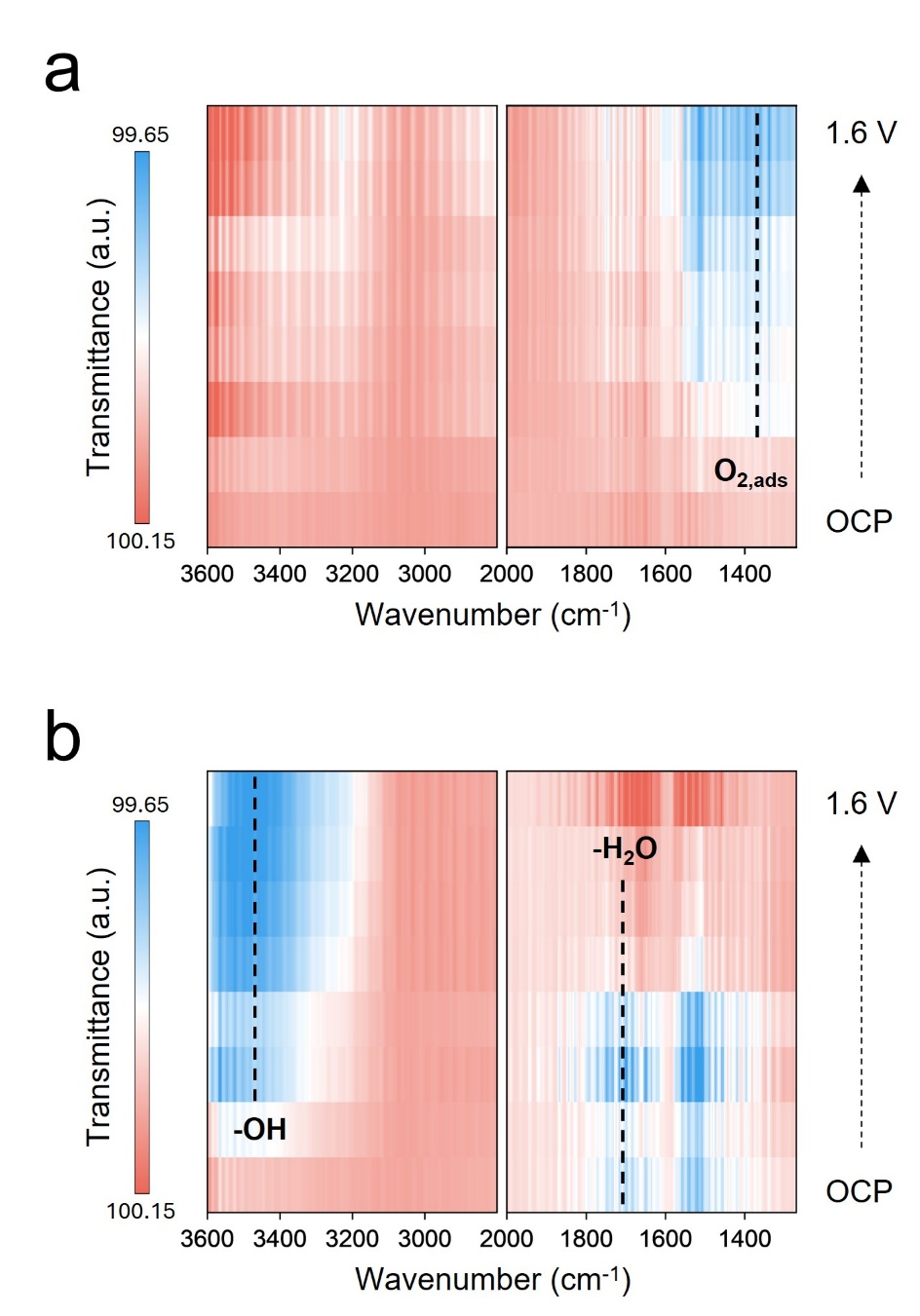


**Figure S13.** In-situ ATR-IR measurements of (a) PM-350, and (b) PM-450 from 1300 cm^-1^ to 3600 cm^-1^.


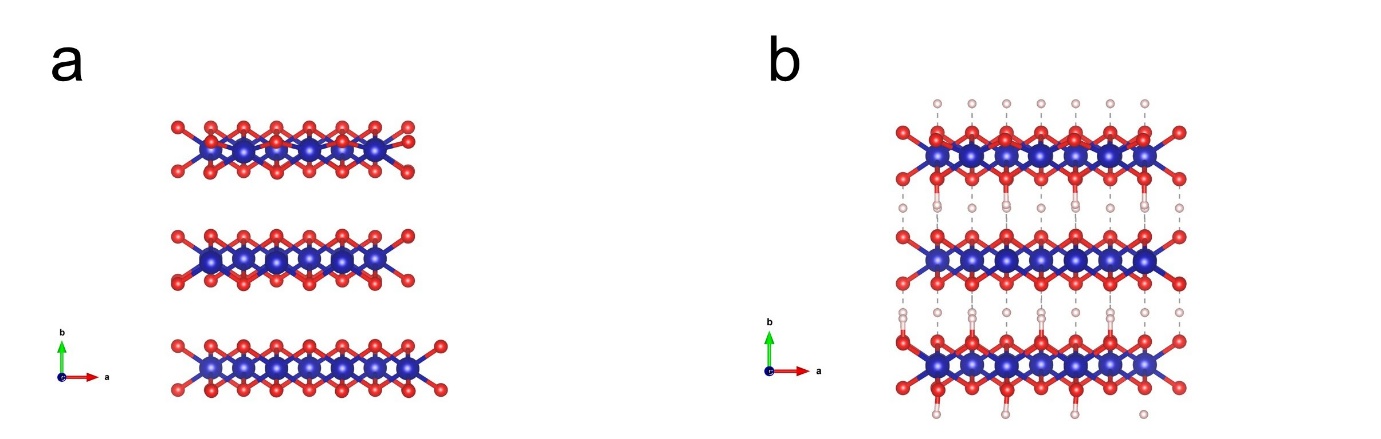


**Figure S14.** The optimized structure for (a) γ-CoOOHₓ (x < 1) and (b) β-CoOOHᵧ (y < 1).


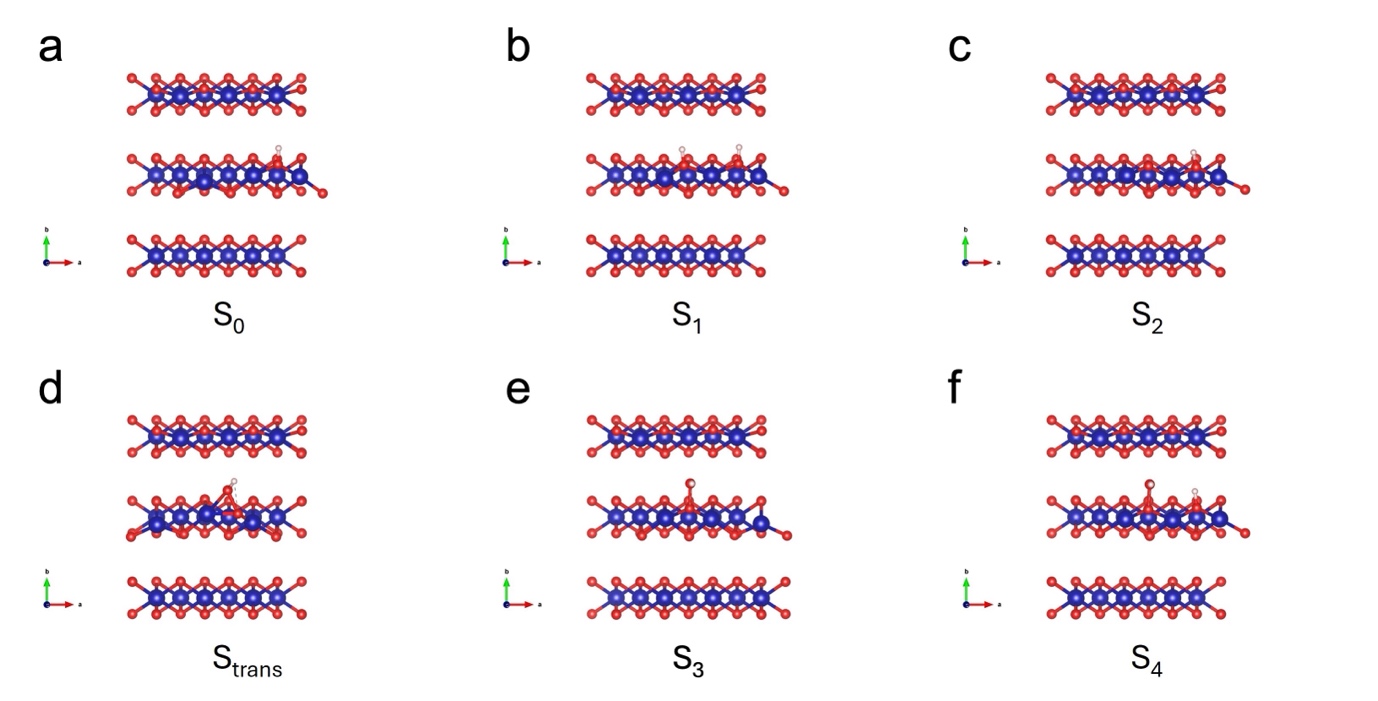


**Figure S15.** The optimized structure for each OER intermediate of γ-CoOOHₓ (x < 1).


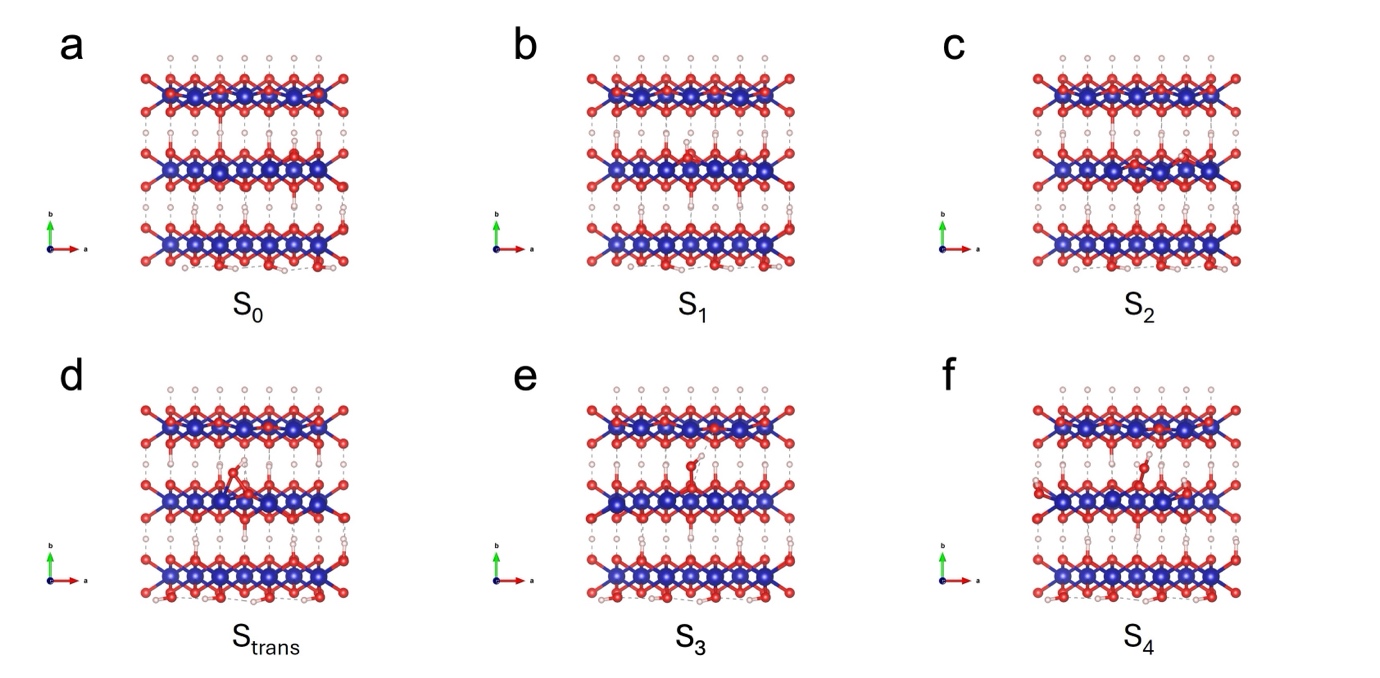


**Figure S16.** The optimized structure for each OER intermediate of β-CoOOHᵧ (y < 1).


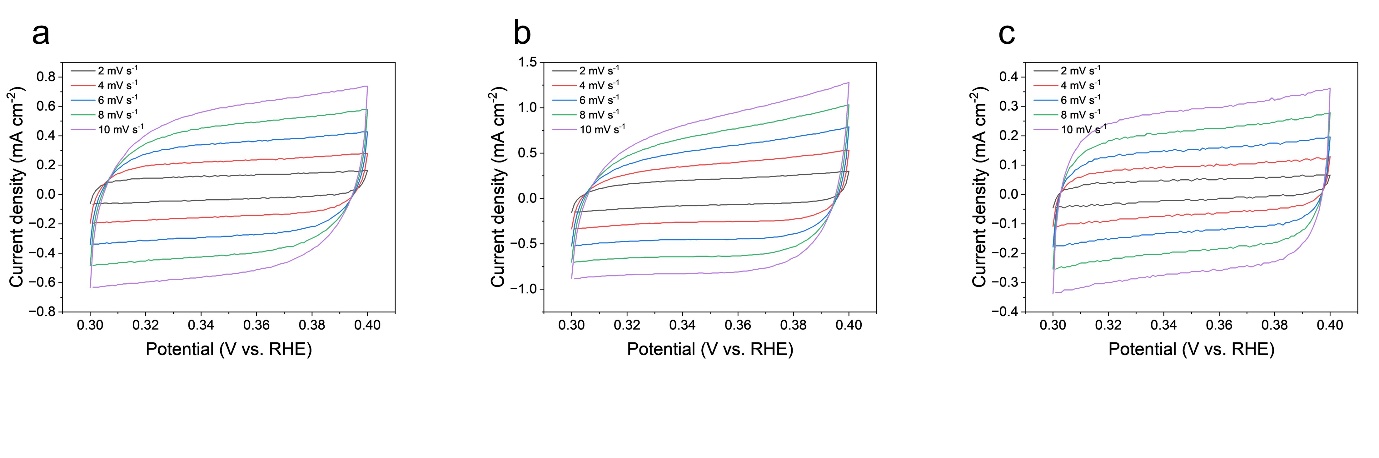


**Figure S17.** CV curves of (a) PM-250, (b) PM-350, and (c) PM-450 acquired at the potential range from 0.3 to 0.4 V vs. RHE.


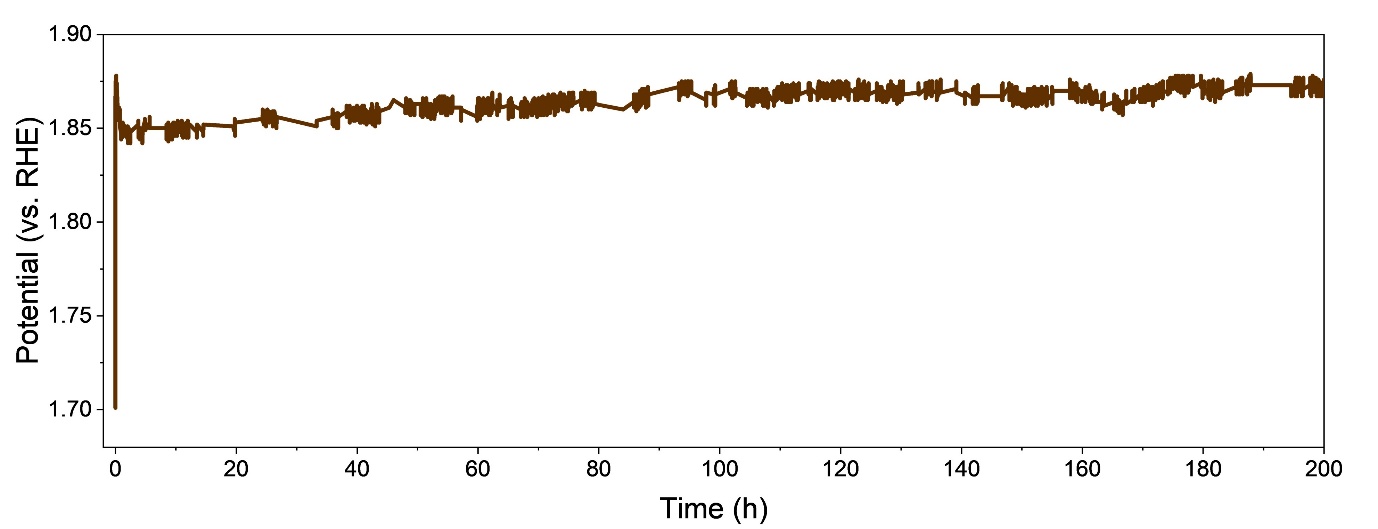


**Figure S18.** Stability test at 500 mA cm^-2^ of the PM-350 sample.


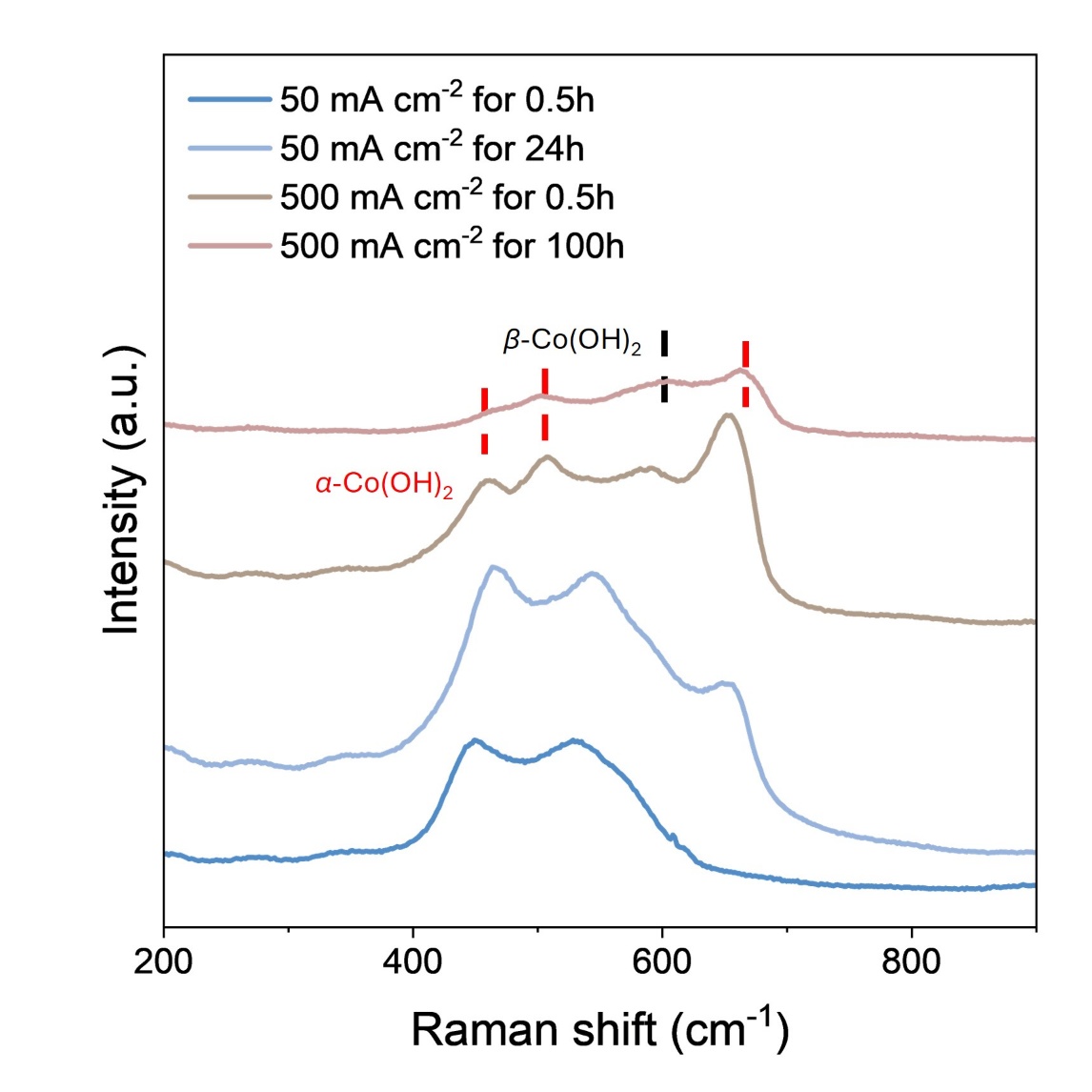


**Figure S19.** Raman test after OER under different conditions.


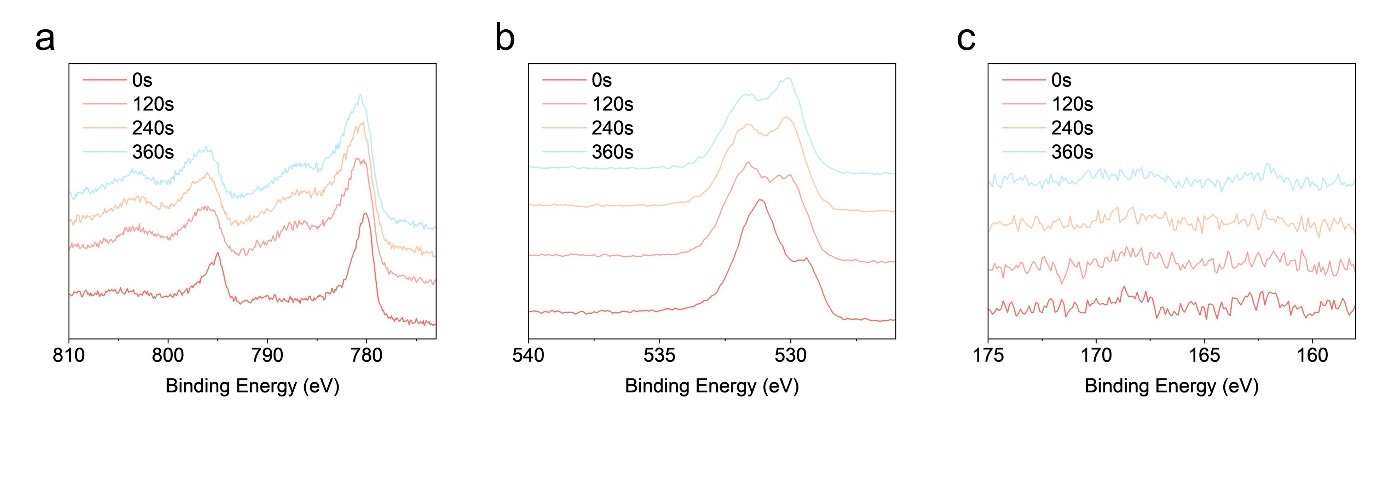


**Figure S20.** In-depth XPS profile of the post-OER samples (50 mA cm^-2^ for 30 min). (a) Co_2p_ spectra, (b) O_1s_ spectra, and, (c) S_2p_ spectra.


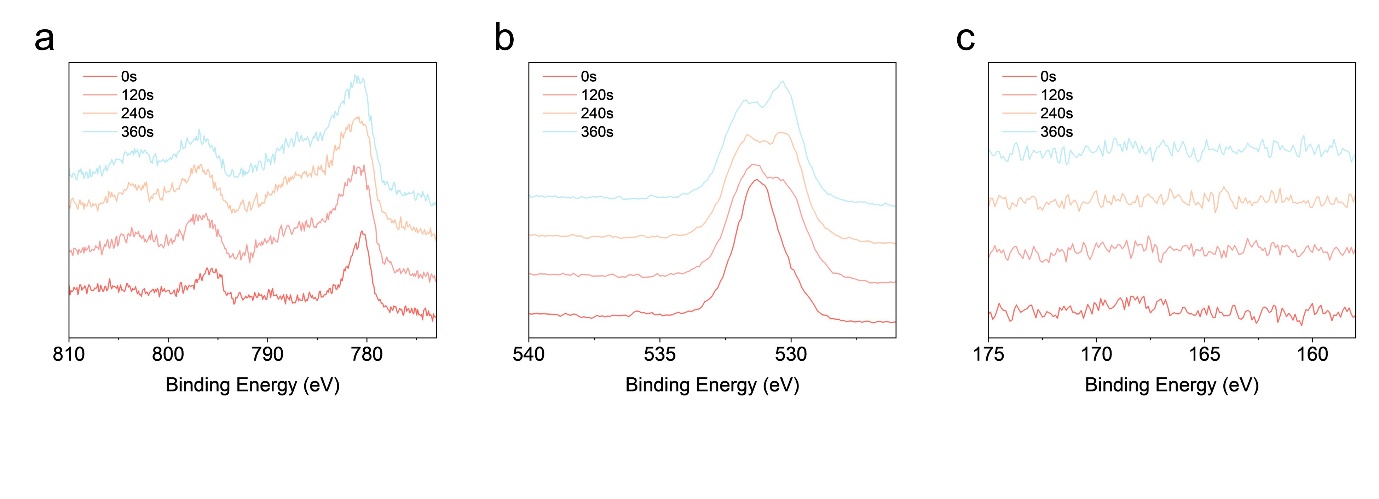


**Figure S21.** In-depth XPS profile of the post-OER samples (500 mA cm^-2^ for 30 min). (a) Co_2p_ spectra, (b) O_1s_ spectra, and, (c) S_2p_ spectra.

Table S1:

| No. | Catalyst | Overpotential  (10 mA cm^-2^) | References |
| --- | --- | --- | --- |
| 1 | MnGa_4_/NF | 293mV | Angew. Chem. Int. Ed **2019**, 58, 16569^[14]^ |
| 2 | Co/VN | 320 mV | Nano Energy **2017**, 34, 1^[15]^ |
| 3 | Co_0.5_(V_0.5_) | 282 mV | Adv. Energy Mater. **2020**, 10, 1903571^[16]^ |
| 4 | NiFeCe-LDH@CP | 232 mV | Chem. Eng. J. **2023,** 464, 142669^[17]^ |
| 5 | MoNiFe (oxy)hydroxide | 242 mV | Nat. Commun. **2022**, 13 (1), 2191^[18]^ |
| 6 | FeCo‐ Co_4_N/N‐ C | 280 mV | Adv. Mater. **2017**, 29, 1704091.^[19]^ |
| 7 | NiCeO_x_-Au | 271 mV | Nat. Energy **2016**, 1, 16053^[20]^ |
| 8 | Sr_3_NiFeMoO_9_-δ | 260 mV | ACS Energy Lett. **2022**, 8, 565^[21]^ |
| 9 | Fe-NiO/NiS_2_ | 270 mV | Angew. Chem., Int. Ed. **2022**, 61, e202207217^[22]^ |
| 10 | Co_3_O_4_-V_Co_ | 262 mV | J. Am. Chem. Soc. **2023**, 145, 2271^[23]^ |
| 11 | (NiCo)S_1.33_ | 302 mV | Nat. Commun. **2023**, 14, 1949^[24]^ |
| 12 | ZnNiCoFeMn HEO_s_-O_v_ | 284 mV | Adv. Funct. Mater. **2024**, 34, 2314820^[25]^ |
| 13 | This work | 267 mV |  |

**Reference:**

[1] Shi, Q.; Chen, Z.; Song, Z.; Li, J.; Dong, J. Angew. Chem. Int. Ed. 2011, 123, 698.

[2] Wu, R.; Qian, X. Zhou, K.; Wei, J.; Lou, J.; Ajayan, P. M. ACS Nano 2014, 8, 6297.

[3] Chen, Y. Z.; Wang, C.; Wu, Z. Y.; Xiong, Y.; Xu, Q.; Yu, S. H.; Jiang, H. L. Adv. Mater. 2015, 27, 5010.

[4] G. Kresse, J. Furthmüller, Comput. Mater. Sci. 1996, 6, 15.

[5] P. E. Blochl, Phys. Rev. B 1994, 50, 17953.

[6] G. Kresse, J. Furthmüller, Phys. Rev. B 1996, 54, 11169.

[7] J. P. Perdew, K. Burke, M. Ernzerhof, Phys. Rev. Lett. 1996, 77, 3865.

[8] Z. Wang, W. A. Goddard, H. Xiao, Nat. Commun. 2023, 14, 4228.

[9] S. Grimme, J. Antony, S. Ehrlich, H. Krieg, J. Chem. Phys. 2010, 132, 154104.

[10] J. K. Nørskov, J. Rossmeisl, A. Logadottir, L. Lindqvist, J. R. Kitchin, T. Bligaard, H. Jonsson, J. Phys. Chem. B 2004, 108, 17886.

[11] A. A. Peterson, F. Abild-Pedersen, F. Studt, J. Rossmeisl, J. K. Nørskov, Energy Environ. Sci. 2010, 3, 1311.

[12] G. Henkelman, B. P. Uberuaga, H. Jónsson, J. Chem. Phys. 2000, 113, 9901.

[13] K. Momma, F. Izumi, J. Appl. Crystallogr. 2011, 44, 1272.

[14] P. W. Menezes, C. Walter, J. N. Hausmann, R. Beltrán-Suito, C. Schlesiger, S. Praetz, V. Yu. Verchenko, A. V. Shevelkov, M. Driess, Angew. Chem. Int. Ed. 2019, 58, 16569.

[15] X. Peng, L. Wang, L. Hu, Y. Li, B. Gao, H. Song, C. Huang, X. Zhang, J. Fu, K. Huo, P. K. Chu, Nano Energy 2017, 34, 1.

[16] K. Fan, H. Zou, L. Duan, L. Sun, Adv. Energy Mater. 2020, 10, 1903571

[17] Y. Y. Liao, R. C. He, W. H. Pan, Y. Li, Y. Y. Wang, J. Li, Y. X. Li, Chem. Eng. J. 2023, 464, 142669, 142669.

[18] Z. He, J. Zhang, Z. Gong, H. Lei, D. Zhou, N. Zhang, W. Mai, S. Zhao, Y. Chen, Nat. Commun. 2022, 13, 2191.

[19] X. Zhu, T. Jin, C. Tian, C. Lu, X. Liu, M. Zeng, X. Zhuang, S. Yang, L. He, H. Liu, S. Dai, Adv. Mater. 2017, 29, 1704091.

[20] J. W. D. Ng, M. Garcia-Melchor, M. Bajdich, P. Chakthranont, C. Kirk, A. Vojvodic, T. F. Jaramillo, Nat. Energy 2016, 1, 16053, 16053.

[21] A. K. Tomar, U. N. Pan, N. H. Kim, J. H. Lee, ACS Energy Lett. 2022, 8, 565.

[22] N. Zhang, Y. Hu, L. An, Q. Li, J. Yin, J. Li, R. Yang, M. Lu, S. Zhang, P. Xi, C. H. Yan, Angew. Chem., Int. Ed. 2022, 61, e202207217

[23] R. Zhang, L. Pan, B. Guo, Z. F. Huang, Z. Chen, L. Wang, X. Zhang, Z. Guo, W. Xu, K. P. Loh, J. J. Zou, J. Am. Chem. Soc. 2023, 145, 2271

[24] Y. Hu, Y. Zheng, J. Jin, Y. Wang, Y. Peng, J. Yin, W. Shen, Y. Hou, L. Zhu, L. An, M. Lu, P. Xi, C.-H. Yan, Nat. Commun. 2023, 14, 1949.

[25] Y. Liu, C. Ye, L. Chen, J. Fan, C. Liu, L. Xue, J. Sun, W. Zhang, X. Wang, P. Xiong, J. Zhu, Adv. Funct. Mater. 2024, 34, 2314820.
